# Mammals show distinct functional gut microbiome dynamics to identical series of environmental stressors

**DOI:** 10.1128/mbio.01606-23

**Published:** 2023-08-31

**Authors:** Adam Koziol, Iñaki Odriozola, Aoife Leonard, Raphael Eisenhofer, Carlos San José, Ostaizka Aizpurua, Antton Alberdi

**Affiliations:** 1 Center for Evolutionary Hologenomics, Globe Institute, University of Copenhagen, Copenhagen, Denmark; 2 Biodonostia Health Research Institute, Donostia-San Sebastian, Spain; University of Hawaii at Manoa, Honolulu, Hawaii, USA; Northwestern University, Evanston, Illinois, USA

**Keywords:** acclimation, adaptation, apodemus, beta diversity, crocidura

## Abstract

The ability of the gut microbiome has been posited as an additional axis of animals’ phenotypic plasticity. However, whether and how such plasticity varies across hosts with different biological features remains unclear. We performed a captivity experiment to compare how the taxonomic, phylogenetic, and functional microbial dynamics varied across a series of temperature and dietary disturbances in two mammals: the insectivorous-specialist *Crocidura russula* and the omnivorous-generalist *Apodemus sylvaticus*. Combining genome-resolved metagenomics, metabolic pathway distillation and joint species distribution modeling, we observed that, although microbiome alpha diversity of both species remained stable, *C. russula* exhibited substantially higher variability and directionality of microbial responses than *A. sylvaticus*. Our results indicate that the intrinsic properties (e.g., diversity and functional redundancy) of microbial communities coupled with physiological attributes (e.g., thermal plasticity) of hosts shape the taxonomic, phylogenetic, and functional response of gut microbiomes to environmental stressors, which might influence their contribution to the acclimation and adaptation capacity of animal hosts.

**IMPORTANCE:**

In our manuscript, we report the first interspecific comparative study about the plasticity of the gut microbiota. We conducted a captivity experiment that exposed wild-captured mammals to a series of environmental challenges over 45 days. We characterized their gut microbial communities using genome-resolved metagenomics and modeled how the taxonomic, phylogenetic, and functional microbial dynamics varied across a series of disturbances in both species. Our results indicate that the intrinsic properties (e.g., diversity and functional redundancy) of microbial communities coupled with physiological attributes (e.g., thermal plasticity) of hosts shape the taxonomic, phylogenetic, and functional response of gut microbiomes to environmental stressors, which might influence their contribution to the acclimation and adaptation capacity of animal hosts.

## INTRODUCTION

The gut microbiome has been posited to confer animals with an increased capacity to tackle environmental variation (1 – 3). To date, there have been studies that have demonstrated how gut microbiomes can confer host-specific functions, such as cold adaptation in mice (4), fat metabolism in hibernating bears (5), or heat stress resistance in tadpoles (6). In order to provide adaptive capacity to animals, microbial communities need to be rearranged in ways that provide functional benefits to their hosts, and at a pace that is fast enough to cope with environmental change. The attribute that measures the level of functional genetic variation a microbiome undergoes in response to disturbances has been termed “metagenomic plasticity” (1, 7). How this attribute varies within and between host species remains unexplored, because the understanding of the basis of metagenomic plasticity requires going beyond mere characterisation of microbial compositions (8 – 11). Here, we address it through modeling the functional dynamics of microbiomes within and between host species using genome-resolved metagenomics.

Metagenomic plasticity can be beneficial for the host, particularly when directional responses toward different disturbances assist animals in adapting to novel conditions (12, 13). However, perturbations to microbial composition and function may also change along neutral expectations with no discernible impact on the host (14, 15), or can also even have negative implications for host fitness, as shifting gut microbiomes toward alternative states can potentially disrupt microbiome-encoded functions that are important for the host (16 – 18). Ascertaining how functional microbiota dynamics vary across host species is an essential step toward understanding whether and how gut microorganisms can contribute to the capacity of animals to acclimate and adapt to environmental variation. Features such as dietary niche (i.e., carnivorous vs herbivorous) or evolutionary background (i.e., Eulipotyphla vs Rodentia) can shape intrinsic microbiota features that can affect metagenomic plasticity. For example, it has been shown that microbial diversity and functional redundancy can buffer perturbations by mitigating functional loss and securing important metabolic interaction networks (19, 20). Hence, from a comparative perspective, microbiomes with higher diversity and redundancy may be less plastic, while microbiomes with lower diversities may undergo larger variation.

To gain insight into how the gut microbiomes in wild organisms respond to multiple disturbances, we conducted a captivity experiment, which exposed two small mammal species with contrasting ecological and microbial characteristics to a series of environmental and dietary challenges. Over a period of 45 days, animals underwent heat exposure, cold exposure, and a dietary shift, all of which have been previously demonstrated to induce gut microbiome shifts in other species (21 – 23). By analyzing fecal samples collected throughout the experiment, we quantified the metagenomic plasticity of two mammal species with different dietary and microbiota features: the omnivorous, generalist mouse *Apodemus sylvaticus* (herein AS), with a higher microbial diversity, and the insectivorous, specialist shrew *Crocidura russula* (herein CR) that was found to harbor a less diverse gut microbiome (24). Based on the contrasting ecological and microbiota traits of both species, we hypothesized that (i) AS would exhibit a more functionally complex and redundant gut microbiome than CR, (ii) that this property would result in more stable microbiome dynamics in AS and more variable in CR in response to environmental variation, and (iii) that those microbiome changes would be more directional (rather than stochastic) in CR than in AS, with stronger associations between microbial composition and metagenomic functions in the experimental treatments. To address these hypotheses, we (i) reconstructed and annotated the bacterial genomes of the gut microbiome of both species, (ii) quantified the variability of the microbiome (in terms of neutral, phylogenetic and functional alpha, and beta diversity) over time in response to the experimental treatments, (iii) modeled the directional temporal trajectories of taxa and associated functions in response to the experimental treatments, and (iv) contrasted the observed patterns between the two host species. Our study represents the first comparison of the functional microbiome dynamics of two species experimentally exposed to a concatenation of environmental stressors. Our results show that functional microbiome dynamics can vary dramatically between host species, and we discuss the ecological implications of the observed differences.

## MATERIALS AND METHODS

### Animal capture

Adult AS (*N* = 22) and CR (*N* = 29) were collected in 11 field sites located in Atlantic forest and meadow habitats in the Northern Iberian Peninsula, Europe (43.2 N, 2.2 W), between June and August 2019. Animals were captured using Sherman traps over a 3-day period at each site and checked every 12 h. Baits used were a mixture of oats and tuna, and a small wedge of apple. Upon successful detection, each animal was transferred into a plastic bag for species and sex identification. Immediately after, individuals were placed into a small microisolator cage for transfer to the ZIBA Animal Experimentation Facility in Zarautz, Basque Country. All animal captures and animal experimentation were approved by the Regional Government of Gipuzkoa under license codes PRO-AE-SS-206 and PRO-AE-SS-168, and performed in accordance with the agreed-upon guidelines and regulations.

### Tagging

Animals were uniquely tagged with a Mini HPT10 RFID chip (Biomark, USA) implanted to the suprascapular region upon entry to the experimentation facility. Animals were first isolated and weighed upon being transferred to an induction chamber connected to an oxygen flowmeter, where they were anaesthetized with 2.5% isoflurane. Animals were removed from the induction chamber and re-administered 1% isoflurane through an attached facemask while implanting the tag. After the successful implantation of the RFID chip, biometrics were taken (head-body length, tail length, and weight), and animals were monitored for 10 min before placing them in their assigned cage.

### Housing conditions

Animals were co-housed with conspecifics of the same sex inside an HPP750Life climate chamber (Memmert). Cage membership was uneven due to variable field capture successes and some mortality of individuals before the experimental conclusion. To minimize losses in environmental microbial access within the external environment, each cage was given a combination of sticks and stones sourced from the local environment as enrichment elements (24) (Fig. S1) and kept at a constant temperature of 20°C and 60% humidity with a 12 h light and dark cycle. Bedding was changed at the end of each experimental treatment. Baseline diets consisted of a commercially available mouse chow for AS (Teklad Global 14%) and gelatinous kitten feed (Royal Canin Kitten 12 months) for CR. Drinking water was provided *ad libitum* and food was added every 24 h.

### Experimental design

**Fig 1:**
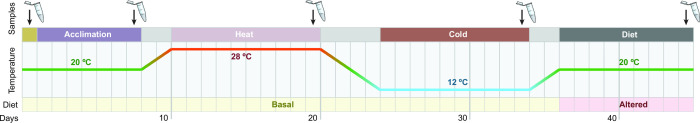
Experimental design. Wild-captured *Apodemus sylvaticus* and *Crocidura russula* were exposed to a concatenation of experimental treatments. Animals were isolated for collecting individual feces at the end of each treatment.

Microbiome variation of CR and AS was induced by exposing individuals to a series of four experimental treatments (Fig. 1) previously known to cause significant microbial perturbations (21 – 23). In the experiment, each treatment consisted of a 10-day exposure, with a 2-day ramp period between treatments (i.e., ambient temperature was gradually modified to the targeted temperature), however during the heat-to-cold transition (28–12°C), this ramp period was extended to 4 days to avoid sudden temperature changes. The diet treatment involved a change in the percentages of protein, fats, and fiber compared to the original formulations. Acclimation conditions represented the baseline conditions, where the ambient temperature was maintained at 20°C and humidity at 60%. Light and dark cycles were not changed throughout the experiment. As the primary focus of the study was to reveal differences in the overall gut microbial dynamics between the two species, and given the logistical and ethical limitations (e.g., access to a single climate chamber, permit to sample a limited number of animals), we chose to not use constant-environment controls as a point of comparison. Finally, fecal samples were collected from each individual on the last day of each disturbance treatment (*n* = 5 per individual).

### Sample collection

Fecal samples (~50 mg) were collected on the final day of each treatment. Each animal was isolated in a sterile cage to obtain fresh uncontaminated samples from each individual at each sampling point. Individuals were checked every 15 min to ensure feces were collected right after defecation. The samples were immediately transferred into 2 mL conical tubes containing 500 µL of DNA/RNA Shield (ZYMO, USA) and stored at −20°C until further processing.

### DNA extraction

Fecal samples were extracted using the in-house developed DREX protocol [for full details, see reference (16)]. In short, 500 µL of feces diluted in the preservation buffer was bead-beaten for cell lysis in 2 mL e-matrix tubes (MP Biomedical, USA) for 10 min, with the position of samples being changed four times using a Tissuelyzer II (Qiagen, Germany). Subsequently, 200 µL of the supernatant was aliquoted into 96 deep-well plates, and the DNA was purified following the bead-based nucleic acid extraction protocol with a final elution of 50 µL. Sample layouts were initially randomized to minimize batch effects, and DNA extraction blanks were included at the beginning of the extraction. These blanks consisted of the same preservation buffer that was used for sample storage and were included throughout all laboratory steps.

### Library quantification and sonification

Extracts were immediately quantified using Qubit (Thermo Fisher, USA), and 400 ng of extract was taken for further processing. The aliquoted samples were fragmented into 320–420 bp-long fragments using a Covaris LE220R ultrasonicator machine (Covaris, USA), and this was confirmed by a high-sensitivity chip on a TapeStation (Agilent, USA). Fragmented DNA was then prepared for BGI sequencing through the ligation of customized blunt-end adapters using the BEST single-tube protocol (25) and purified using a bead-purification method. Subsequent libraries were purified and unique dual-indexed primers were added through indexing PCR. The indexing PCR was carried out in 50 µL reactions, consisting of 10 µL template DNA, 25 mM dNTPs, 10 µM forward and reverse primers, 10× buffer, 25 mM MgCl_2_, five units TagGold, and ddH_2_O. After one last purification, libraries were quantified using a fragment analyzer and pooled equimolarly. All samples were sequenced on a DNBSEQ 150 × 150 bp paired-end flow cell (BGI, China).

### Bioinformatic processing

Raw, demultiplexed reads were processed using an in-house developed pipeline available in Github (see Data Availability Statement). In brief, we prepared the reads by first trimming the sequencing adapters and removing low-quality and short reads using fastp v0.23.1 (26). We removed host DNA from the metagenomic samples by mapping the fastp processed reads to host reference genomes using Bowtie2 v2.4.4 (27). Due to the unavailability of a reference genome for CR, we used a close relative: *Crocidura indochinensis* (28). BAM files were processed using samtools v1.12 (29). The non-host reads were then co-assembled by individuals across the time series of samples using megahit (30) and the raw reads were mapped to the contigs using Bowtie2. The contigs were then binned using three binning algorithms, CONCOCT (31), MetaBAT 2 (32), and MaxBin 2 (33), and the final metagenome-assembled genomes (MAGs) were refined using DAStool (34). We only retained MAGs that were at a minimum 70% completeness and less than 10% contamination as estimated using CheckM v1.0.12 (35), to minimize genome completeness biases (36). Dereplication of the associated MAGs was then performed at 98% average nucleotide identity (using ANImf) with dRep v3.3.0 (37). To obtain the coverage statistics for each MAG, we mapped the non-host reads to the list of dereplicated MAGs. The non-host reads from each sample were mapped back to the list of MAGs using Bowtie2 and read counts were calculated using CoverM (https://github.com/wwood/CoverM) to generate a read count table. MAG read count data were normalized for both genome length and sequencing depth by converting them to reads per million (38).

We used GTDB-tk (v2.1.0; database = r207 v2) (39) to place the reconstructed genomes in the reference bacterial tree through phylogenetic placement. The final tree was obtained by pruning the tips of the reference GTDB genomes using the function keep.tip in the R package ape, yielding the phylogenetic tree of our MAGs. Open reading frame prediction, gene calling and functional annotation was performed using DRAM, which include annotation against the Pfam, KEGG, UniProt, CAZY, and MEROPS databases (40). We distilled functional annotations into Metabolic Capacity Indices (MCI) using the R package distillR (github.com/anttonalberdi/distillR), to obtain biologically meaningful annotations indicating the capacity of each MAG to degrade or produce relevant compounds for host metabolism. The reference database included 251 metabolic pathways and modules obtained from the KEGG (41) and Metacyc (42) databases, which were used to convert unprocessed annotations into 136 genome-inferred functional traits (GIFTs) (Table S1). These pathways are used to determine the metabolic capabilities of microorganisms by quantifying the relative abundance of genes necessary to perform specific metabolic tasks. The GIFTs are scored on a scale of 0 to 1, where 0 indicates the absence of all genes related to the pathway and 1 indicates the presence of all of them. If a pathway step requires the presence of two identifiers, it is considered complete when both are present, half-complete when only one is present, and empty if none are present. GIFT values were adjusted for MAG genome completeness to minimize functional biases (43), and GIFTs within each of the 14 analyzed metabolic functions were averaged to obtain the MCI values used for the statistical modeling. All analyses were performed in R v4.2 (44) and the subsequent packages used for analysis are cited below.

### Data analysis

#### Alpha/beta diversities

Diversity analyses were performed using the Hill numbers framework (45). All analyses were performed at an order of diversity (*q* value) of 1, which weighs the MAGs according to their relative abundances, and considering different components of diversity. Neutral and phylogenetic Hill numbers were calculated using hillR (46). Neutral Hill numbers only rely on the relative abundance information to compute diversity, while phylogenetic Hill numbers also incorporate branch-length information of the phylogenetic tree of the MAGs, and the functional Hill numbers account for the functional differences between MAGs, based on a distance matrix derived from distilled functional traits of MAGs (47). Alpha diversities were calculated for each individual at each time point. Beta diversities were calculated using the Sørensen-type turnover (48, 49), and were computed both for subsequent time points, to measure the compositional changes across different time points, as well as between all time points to measure overall variability within an individual through time. Values closer to 1 indicate high compositional differences, whereas values close to 0 indicate less compositional differences. Functional beta diversities were calculated from the MCIs table produced by distillR and the normalized count data. The MCI table containing the functional traits was transformed into a distance matrix using Gower’s distance (50), and beta diversities were represented as the FD_beta score provided by hillR (46). Finally, we calculated functional redundancy within the gut microbiome using the R package adiv (51) in terms of the Rstar value derived from the Ustar generated from the uniqueness function of adiv. The Ustar is calculated as the ratio between Rao quadratic diversity index and the Simpson index, measuring the functional uniqueness of the community. Complementary to the Ustar, the Rstar is calculated as 1 − Ustar and denotes how functionally redundant the community is in relation to a scenario where the community is completely functionally unique (closer to 0).

To analyze variations in alpha and beta diversities in relation to study species and experimental disturbances, we used linear mixed-effect models with the R package nlme (52) (see Table S2 for final models used). To test the null hypothesis of no difference in alpha diversity between species, we used alpha diversity as the response variable and species (categorical factor with two levels: AS and CR) as fixed explanatory variables. As several individuals were maintained in each cage and repeated measures were taken from each individual, we specified random effects with a random intercept of the form “~1|Cage/Individual_ID.” Model assumptions of homoscedasticity and normality of errors were evaluated by visual inspection of residual plots. Additionally, as repeated samples were taken over time, the assumption of independence of residuals was evaluated through the acf() function in R. Model assumptions were evaluated similarly in the rest of the fitted models.

To test the null hypothesis of no difference in beta diversity between species, overall beta diversity within each individual between all time points was used as response variable and species as fixed explanatory variable. In this case, a single value was generated per individual and a random effect of the form “~1|Cage” was used. To analyze the variations in alpha and beta diversities through time we fitted the models separately for AS and CR. To test the null hypothesis of no effect of experimental disturbances on alpha diversity, alpha diversity was used as response variable and experimental disturbance (categorical factor with five levels) as a fixed explanatory variable. Again, we used a random effect of the form “~1|Cage/Individual_ID.” To test the null hypothesis of no effect of experimental disturbances on beta diversity, pairwise beta diversities between consecutive time points were used as response variables. We used the same model structure as in alpha diversity models with one modification: the explanatory variable treatment was replaced with treatment-pairs (categorical factor with four levels), which represents the beta diversity between consecutive time points.

#### Effects of experimental disturbances on microbial composition

To assess the degree of directional compositional changes in the gut microbiome of AS and CR in response to the experimental treatments, we performed PERMANOVA (53) on each of the dissimilarity matrices calculated from the beta diversities metrics using hillR. For that, we used the function ‘adonis2’ in the R package ‘vegan’ (54) with the formula: adonis2 (microbiome-beta diversity ~Treatments, strata = Individual_ID, data = data). The magnitude of the directional change was quantified through the unadjusted *R* ^2^ associated with the experimental disturbances. Finally, to visualize the variation driven by treatment differences, we visualized the systematic patterns in the multivariate data set using a constrained ordination: canonical analysis of principal coordinates (CAP) ( 55) on the same dissimilarity matrix as in the PERMANOVAs. CAP explicitly incorporates group membership information in its analysis, and provides results in the form of canonical axes, which are linear combinations of the dissimilarity matrix. These axes can be interpreted in terms of the contribution of different variables to the separation between groups.

#### Joint species distribution modeling with Hmsc

To further explore the directional response of the gut microbiome to experimental disturbances, we modeled MAG-level (i.e., microbial species) trajectories in response to the experimental treatments, as well as community level responses in metagenomic functional potential, using joint species distribution modeling (56) as implemented in the R package Hmsc (57). Hmsc is a multivariate hierarchical generalized linear model that uses Bayesian inference. In the matrix **Y** of Hmsc, which typically includes the species abundance or occurrence values, we included the sequence abundance of each MAG across sampling units (scaled to 0 mean and unit variance), and then we fitted the log-normal model to each MAG. As data on rare species do not contain sufficient information to fit species-specific models, we only included MAGs that reached 0.1% relative abundance in at least one sampling unit, which resulted in 322 MAGs for the response matrix of the AS models and 54 MAGs for the response matrix of the CR models. The matrix X of Hmsc includes environmental variables to be used as predictors of species distributions, as fixed effects. In the matrix X, we included the categorical factor experimental treatment and the log-transformed variable sequencing depth, which controlled for different sampling efforts between samples. Additionally, to account for the repeated measures within individuals and nested study design, we included individual ID and cage ID as random effects. The T matrix of Hmsc includes functional traits of species, and species responses to the fixed effects (the variables in X matrix) are modeled as a function of functional traits in matrix T. In our case, to examine community-wide functional response to the treatments, we included in the T matrix of the Hmsc the 14 function-level MCIs produced with distillR. These were the metabolic capacity indices for the degradation of alcohol, amino acids, antibiotics, lipids, nitrogen compounds, polysaccharides, sugar and xenobiotics, as well as the biosynthesis of amino acids, amino acid derivatives, aromatic compounds, organic anions, SCFA, and vitamins. Based on their functional characteristics, the final functional trait matrix T consisted of a value between 0 and 1 denoting the estimated capacity of each MAG to perform each metabolic function. We reported the results of community-wide functionality, as the predicted community-weighted mean capacity to fully complete each metabolic function, together with the predicted posterior 90% credible intervals. Non-overlapping posterior credible intervals were interpreted as strong evidence for significant temporal change in functional capacity. We also included the MAG phylogeny in the matrix **C** to quantify the degree of phylogenetic signal in the MAG’s responses to the treatments. Hmsc models the species’ responses to the environmental predictors as a function of their functional traits, so that functionally similar species are expected to respond similarly to the predictors in X matrix. The phylogenetic signal measures the tendency of closely related species to respond similarly to the environmental variables, once their functional similarities (based on the traits in **T** matrix) were taken into account. The phylogenetic signal is measured by the Rho parameter which takes any values between 0 and 1, with 0 indicating no phylogenetic signal in MAG’s responses to the treatments and 1 indicating completely phylogenetically structured responses, based on the Brownian model of trait evolution. Strong phylogenetic signal, not captured by the traits included in the T matrix, is usually interpreted as an effect of phylogenetically structured traits that were not included in the model. We fitted the models assuming default priors and sampled the posterior distribution running four Markov Chain Monte Carlo (MCMC) chains each of which was run for 3,750 iterations with 1,250 discarded as burn-in. We thinned by 10 to obtain a total of 250 posterior samples per chain and 1,000 total posterior samples. To test for MCMC convergence we measured the potential scale reduction factor (57 ) for the beta (response to perturbations), gamma (trait response to perturbations), and rho parameters (phylogenetic signal).

## RESULTS

**Fig 2:**
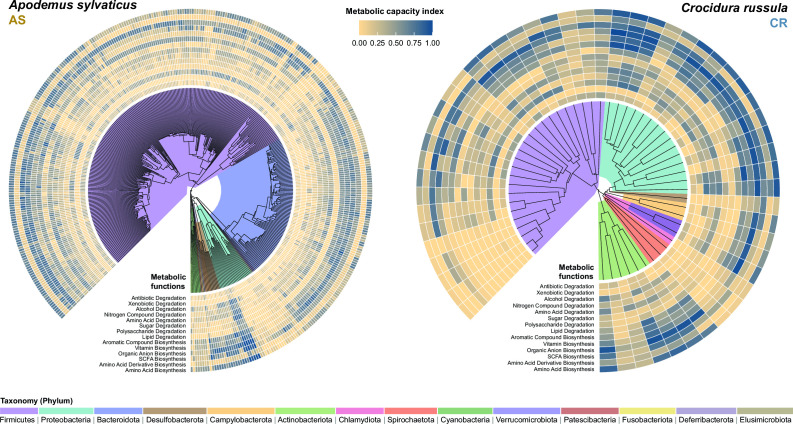
Radial tree visualizing the phylogenetic relationship for each high- and medium-quality MAGs, with outer rings indicating their functional capacity to perform each of the 14 metabolic functions to biosynthesis or degrade biomolecular compounds. Metabolic capacity index (MCI) refers to the average value of the relative proportion of genes present in each genome to biosynthesize or degrade multiple compounds within the metabolic function. Values closer to 1 (dark blue) indicate a higher capacity, while values closer to 0 (light yellow) indicate lower or no capacity.

We generated a total of 2,556,840,654 (mean = 15,040,239 ± 6,609,535) and 1,590,530,664 (mean = 15,040,239 ± 14,021,857) reads from 105 and 120 fecal samples of 22 AS and 29 CR individuals, respectively (see Supplementary Data 1 for sample information). The genome-resolved metagenomic analysis yielded 412 (AS) and 54 (CR) MAGs of adequate quality. Based on the criteria established in Bowers et al. (58), 278 and 43 MAGs were considered high-quality and 134 and 11 MAGs were considered medium-quality for AS and CR, respectively (Supplementary Data 2). The microbiome of AS was dominated by Firmicutes and Bacteroidota, while the Firmicutes and Proteobacteria were dominant in CR (Fig. 2). The functional profiles of the bacteria of both microbial communities also exhibited marked differences (Fig. S2).

### Microbial diversity and functional redundancy of AS and CR

**Fig 3:**
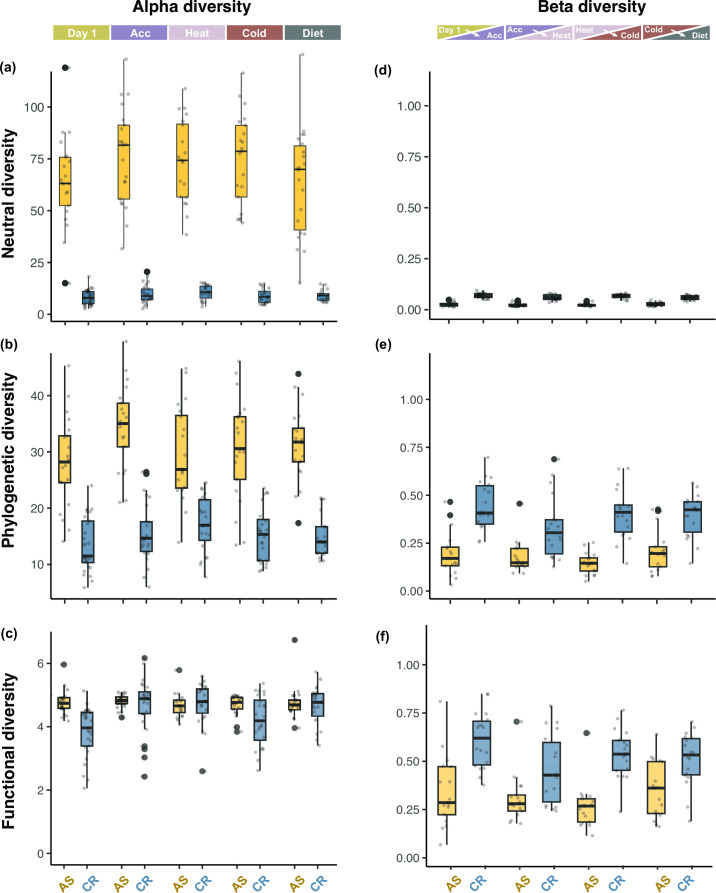
Alpha and beta diversity metrics. (**a–**c) Hill numbers (order of diversity 1) for *Apodemus sylvaticus* (AS, yellow) and *Crocidura russula* (CR, blue) showing neutral, phylogenetic, and functional alpha diversities. (**d–**f) Hill numbers (order of diversity 1) for AS and CR showing neutral, phylogenetic, and functional beta diversities, computed as Sørensen-type turnover metrics.

**Fig 4:**
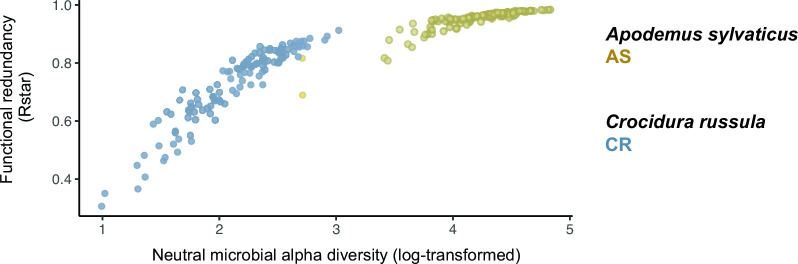
Relationship between the logarithmic value of neutral microbial diversity (order of diversity 1) and the calculated functional redundancy for *Apodemus sylvaticus* (yellow) and *Crocidura russula* (blue).

Comparatively, between the two species, AS harbored a significantly larger effective number of MAGs than CR in terms of neutral (D_N_) (*F*_1, 9_ = 804.403, *P* ≤ 0.001), phylogenetic (D_P_) (*F*_1, 9_ = 90.269, *P* ≤ 0.001), and functional (D_F_) (*F*_1, 9_ = 5.342, *P* = 0.046) alpha diversities (Fig. 3). Accordingly, we detected a significantly higher level of functional redundancy in AS compared to CR (*D =* 0.917, *P* < 0.001). Furthermore, a strong positive relationship was observed between diversity and functional redundancy, particularly in CR (Fig. 4). However, across all samples of AS, the functional redundancy remained high, mainly due to the high number of phylogenetically and functionally similar Firmicutes strains.

### Temporal variations of the gut microbiomes in response to experimental disturbances in AS and CR

Partitioning out the different components of alpha diversity revealed that each host species responded differently to each of the diversity metrics. With regards to neutral diversity, we found no evidence of significant changes in the neutral MAG diversity for either species (AS_N_: *F*_4, 79_ = 1.762, *P* = 0.144; CR_N_: *F*_4, 87_ = 1.588, *P* = 0.182). However, for phylogenetic alpha diversity, we found moderate evidence of temporal change in AS and weak evidence in CR (AS_p_: *F*_4, 79_ = 3.129, *P* = 0.019; CR_p_: *F*_4, 87_ = 2.046, *P* = 0.095) (Fig. 3a and b). Regarding functional alpha diversity, we found no evidence of temporal changes in AS and significant evidence in CR (AS_f_: *F*_4, 79_ = 1.051, *P* = 0.386; CR_f_: *F*_4, 87_ = 7.124, *P* = 0.001).

CR showed significantly higher overall beta diversity compared to AS in terms of taxonomically neutral (*F*_1, 9_ = 21.996, *P* = 0.001), phylogenetic (*F*_1, 9_ = 81.003, *P* = <0.001), and functional (*F*_1, 9_ = 20.782, *P* = 0.001) beta diversities (Fig. 3d through f), indicating that the CR microbiome varied more over time compared to the AS microbiome. Additionally, treatment-pairs did not have a significant effect on the beta diversity of AS, meaning that AS microbiome changed at a similar rate over time, regardless of the treatments (Fig. 3d through f). In contrast, in CR, we observed a significant variation in beta diversity across treatment-pairs for taxonomically neutral diversity (CR_N_: *F*_3, 51_ = 3.762, *P* = 0.016) and, particularly, for functional beta diversity (CR_F_: *F*_3, 51_ = 3.308, *P* = 0.027), but not for phylogenetic beta diversity (CR_P_: *F*_3, 51_ = 2.167, *P* = 0.103).

### Directional effect of experimental disturbances on gut microbiome composition of AS and CR

**Fig 5:**
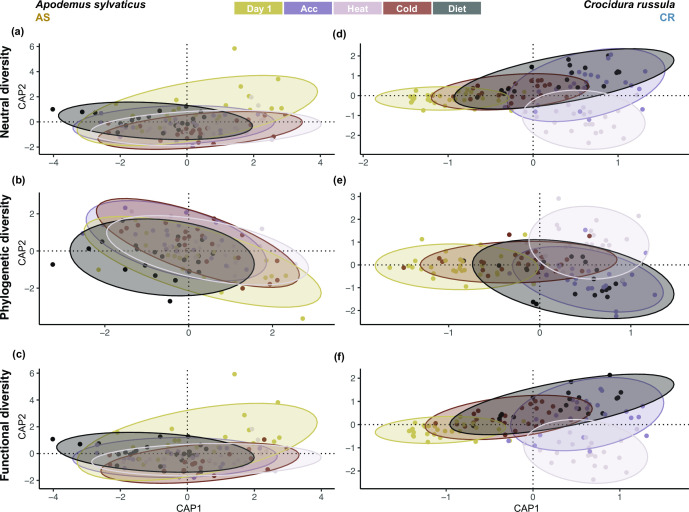
Canonical analysis of principal components plot constrained to each disturbance on the gut microbiome composition of *Apodemus sylvaticus* and *Crocidura russula* using the Sørensen-type turnover, performed for (**a and **b) neutral, (**c and **d) phylogenetic, and (**e and **f) functional diversity metrics. Samples are colored by experimental disturbance and the distance between points represents the dissimilarity in microbial assemblage.

The directional effect of the experimental disturbances on microbiome composition was markedly higher in CR than in AS (Fig. 5). This was demonstrated when composition was measured in terms of neutral (AS_N_: *R* ^2^ = 0.039, *P* = 0.007; CR_N_: *R* ^2^ = 0.298, *P* ≤ 0.001), phylogenetic (AS_P_: *R* ^2^ = 0.074, *P* = 0.005; CR_P_: *R* ^2^ = 0.28, *P* ≤ 0.001) as well as functional indices (AS_F_: *R* ^2^ = 0.039, *P* ≤ 0.001; CR_F_: *R* ^2^ = 0.301, *P* ≤ 0.001), where the explanatory power of the PERMANOVA was more than four times higher for CR than for AS.

**Fig 6:**
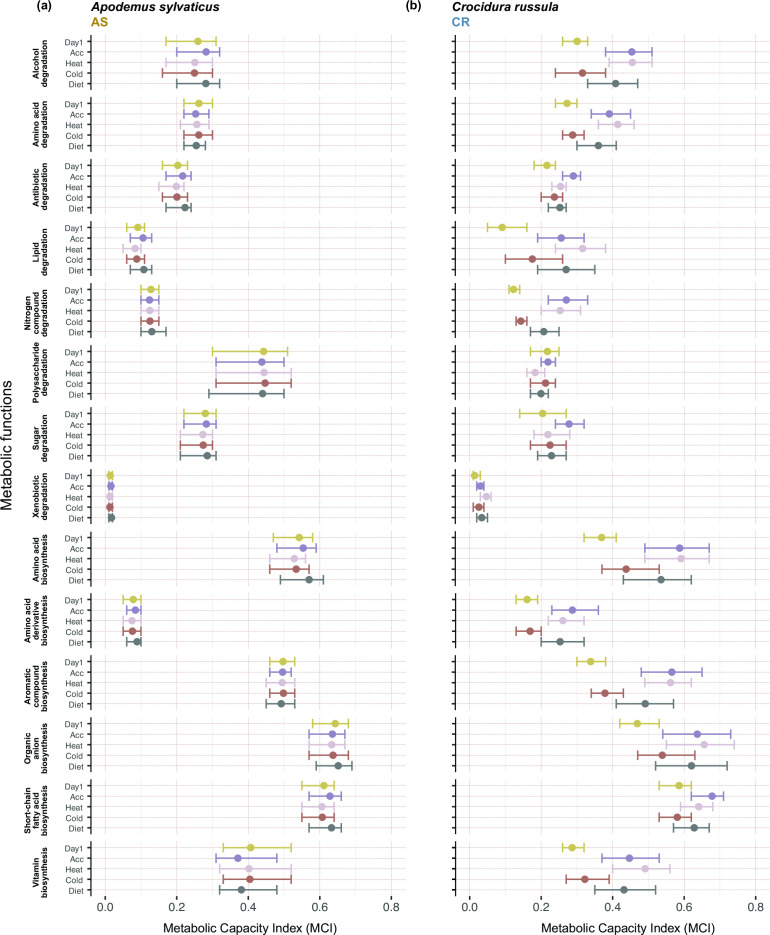
Predicted functional capacities from 14 functional Metabolic Capacity Indices (MCIs) for *Apodemus sylvaticus* and *Crocidura russula* representing different degradation and biosynthesis capacities. Points indicate the community-weighted mean capacity to fully complete each metabolic function. The error bars depict 90% credible intervals; non-overlapping posterior intervals were interpreted as strong evidence for significant difference in community-weighted mean capacity between time points.

MAG-level analyses through joint species distribution modeling revealed that the directional responses of MAGs to the treatments in the AS gut microbiome did not result in differences in community-level functional capacities for the host-relevant functions studied, as indicated the by the broadly overlapping posterior credible intervals (Fig. 6). Conversely, several functions in CR gut showed variations across treatments: the community-wide capacities for lipid degradation, nitrogen compound degradation, SCFA production, organic anion production, amino acid production, amino acid derivative production, and vitamin production tended to increase during acclimation and heat treatments, then reduced with the cold treatment, and recovered again during diet treatment (Fig. 6). These functional changes were strongly associated with the abundance dynamics of a *Providencia alcalifaciens* (Enterobacteriaceae) strain with high capacities to perform these metabolic functions (Fig. S3). The response of the MAGs to the experimental disturbances had strong phylogenetic signal in both AS (Rho = 1 [0.99, 1]) and CR (Rho = 0.87 [0.65, 0.99]), meaning that phylogenetically related MAGs tended to respond similarly to the treatments.

## DISCUSSION

For the first time, we utilized genome-resolved metagenomics in two wild-caught mammals, *Apodemus sylvaticus* (AS) and *Crocidura russula* (CR), across a sequence of environmental and dietary challenges. Our analyses revealed that AS possesses a more diverse and functionally redundant gut microbiome, with more stable microbial dynamics when compared to CR. Further, the CR gut microbiome underwent directional responses (microbial communities of all animals responded in a similar way) to disturbances which, in turn, translated into significant changes in community-wide capacities to perform several metabolic functions important for host performance.

Despite the tendency to limit diversity and dissimilarity analyses to a single or a few widely employed metrics, such as Shannon diversity or Unifrac distances, combining metrics that account for different components and partitions of diversity has proven effective to understand the complexity of microbial diversity patterns (48, 59). In our case, we observed that while the neutral and phylogenetic alpha diversities in AS were significantly higher than in CR, functional diversity was similar in both species. This pattern indicates that the fewer microorganisms associated with CR were functionally more dissimilar to each other, and also highlights that the use of phylogenetic alpha diversity as proxy for functional diversity might be misleading. Hill numbers provide a unified mathematical framework that not only enables accounting for richness, evenness, phylogenetic and functional components of diversity, but also makes it possible to partition diversity into alpha, beta, and gamma components (48). We found that neutral, phylogenetic, and functional alpha diversity metrics remained rather stable in both AS and CR. In contrast, beta diversities revealed that the community composition oscillated between disturbances. This combined observation indicates that the loss and gain of microbial taxa and functions occurred in a balanced way in both species. However, beta diversity variation was more prominent in the gut microbiome of CR compared to AS (Fig. 3d:f), suggesting that the CR gut microbiome is more sensitive to environmental and dietary disturbances. Taxonomic replacement of MAGs did not have a significant impact on the functional repertoire in AS, but in CR it led to significant changes in the functional capacity of the microbiome. Metabolic pathways related to the nutrition and energy metabolism of insectivorous shews, including amino acid, lipid, nitrogen compound, aromatic compound, and vitamin metabolism, underwent considerable fluctuations (Fig. 6). In contrast, pathways involved in the degradation of polysaccharides sugars and xenobiotic compounds remained constant, probably as a result of their limited relevance in the gut microbiota of animals that do not consume plant material.

The differential response in microbiome dynamics could partly be attributed to the difference in microbial diversity between the two species. AS was characterized by a high-diversity microbiome, which correlated with high stability. In contrast, CR had a significantly less diverse microbiome, which correlated with considerably higher dynamics. Previous studies have demonstrated that many microbial species share a high degree of functional similarity, as core genes encoding housekeeping functions make up the majority of their functional capacity (60, 61). Accessory genes tend to occur at low frequency within individual microbial strains and/or populations, thus contributing to higher functional redundancy in more diverse microbial communities (62). We found a similar result demonstrating that higher diversity microbiomes exhibit larger functional redundancies than lower diversity microbiomes (Fig. 5). This was not only evident between species, with AS harboring significantly higher functional redundancy, but also within species, with individual samples of higher diversity consistently exhibiting higher redundancies (Fig. 4). High functional redundancy has been associated with increased microbial stability and resilience to perturbations in many species (62, 63) and may partly explain the significantly lower functional beta diversities detected in AS. This is largely due to both the overall microbial diversity and the frequency of overlapping functions in the AS gut microbiome (Fig. S2b) which would buffer the consequences of functional loss during community compositional changes.

The higher compositional separation, greater predictive power, and lower inter-individual variation observed in CR indicated their responses to environmental stressors were more directional than those observed in AS, indicating that stressors triggered similar and predictable responses across individuals (64 – 66). All three metrics of diversity (neutral, phylogenetic, and functional) demonstrated similar degrees of directionality within CR, with effect sizes five to nine times higher than AS. For both species, we also detected that the bacterial responses to the treatments were strongly phylogenetically structured. Hmsc models calculate the phylogenetic signal as the residual phylogenetic structure in bacterial species responses to the treatments after accounting for the influence of the functional traits (57). The residual phylogenetic signal may be due to the criteria we used to select functional traits: we selected a set of microbial-encoded pathways that were biologically relevant for the animal host, rather than a comprehensive list of functions relevant to predict the microbial responses to the experimental disturbances. The strong phylogenetic signal suggests that there are other phylogenetically conserved functional characteristics that determine bacterial responses to the treatments, which cause phylogenetically related microbes to respond similarly (67).

Our experiment aimed to assess the overall dynamics of microbiota in response to various environmental disturbances, rather than providing detailed insights into the specific effects of each disturbance. Due to the logistic limitations mentioned in the methods section, we were unable to include control samples without disturbances or collect intermediate sampling points between each disturbance treatment. Consequently, our ability to generate definitive results regarding the impact of individual disturbances on the microbiota of AS and CR is limited. Nevertheless, as we included animals from various localities and a wide range of ages, some noteworthy patterns can be briefly discussed with some confidence. In CR, we observed that overall metabolic capacities of the microbiome exhibited a sharp decrease when dropping temperatures (transition from Heat to Cold treatments) (Fig. 6). Although uncontrolled factors could have driven the changes without stable environmental controls, it is unlikely due to the higher overall predictions of functional traits related to different biosynthesis/degradation processes responding so directionally to temperature. Temperature is known to significantly impact the metabolic potential of many small mammals, especially shrews, whose metabolic rate can be reduced by 30% under cold exposure (68, 69). A reduction of the nutritional intake could have favoured microbial strains with lower energetic requirements, which are often characterized by smaller genomes and lower metabolic capacities (70). Our study found that temperature was able to both increase and decrease the metabolic energetics of the CR gut microbiome by regulating the dynamics of modules involved in many metabolic processes, suggesting that not only the intrinsic properties of the microbiome communities (diversity and redundancy), but also the physiological attributes of hosts, shape the functional dynamics of microbial communities.

### Conclusions

Our results reveal, for the first time, that host species with varying evolutionary, ecological, and microbial traits display substantially distinct gut microbial responses under similar conditions. The higher microbiome plasticity observed in *Crocidura russula* is probably due to its less diverse and redundant microbiome, along with a more adaptable thermoregulation capability of the host, compared to *Apodemus sylvaticus*. The inclusion of functional attributes of bacteria in the microbial community modeling was proven useful to identify contrasting dynamics of metabolic functions, and the differences between neutral, phylogenetic, and functional diversity metrics showcased the importance of evaluating multiple attributes of host-microbial communities. The reported discoveries suggest that further investigations on metagenomic plasticity in a wider range of host species from different taxonomic groups would significantly enhance our knowledge about the influence of microbes on host ecology and evolution.

## Data Availability

All data are publicly available under the Bioproject accession number PRJEB58265. Pipelines and scripts used for data analysis are available in Github repositories. Bioinformatic code: https://github.com/AdamKoziol1992/holoflow.git. Statistical analysis code: https://github.com/AdamKoziol1992/Metgen_plasticityanalysis.git.

## SUPPLEMENTAL MATERIAL

Supplemental material mbio.01606-23-s0001.docx Supplemental tables and figures.

ASM does not own the copyrights to Supplemental Material that may be linked to, or accessed through, an article. The authors have granted ASM a non-exclusive, world-wide license to publish the Supplemental Material files. Please contact the corresponding author directly for reuse.
