# Supplementary material for: Mammals show distinct functional gut microbiome dynamics to identical series of environmental stressors
Source: mBio. 2023 Aug 31;14(5):e01606-23. doi: 10.1128/mbio.01606-23 (PMC10653949; doi:10.1128/mbio.01606-23)

Supplementary Information

**Mammals show distinct gut microbiome dynamics to identical series of environmental stressors**

Supplementary tables

**Supplementary table 1**. Nomenclature, classification and definitions of metabolic pathways employed in the functional distillation analysis.

| **Pathway** | **Element** | **Function** | **Definition** |
| --- | --- | --- | --- |
| D070101 | 2,3-Butanediol | Alcohol degradation | 1.1.1.4,1.1.1.76 |
| D070201 | Ethanol | Alcohol degradation | 1.1.1.1 1.2.1.10 |
| D070202 | Ethanol | Alcohol degradation | 1.1.1.1 1.2.1.3 6.2.1.1 |
| D070401 | Glycerol | Alcohol degradation | 2.7.1.30 1.1.5.3 |
| D070402 | Glycerol | Alcohol degradation | 1.1.1.6 2.7.1.29 |
| D070403 | Glycerol | Alcohol degradation | 4.2.1.30 1.1.1.202 |
| D070404 | Glycerol | Alcohol degradation | 1.1.1.6 2.7.1.121 |
| D070501 | Propylene glycol | Alcohol degradation | 4.2.1.28 (1.1.1.1,(1.2.1.87 2.3.1.222 (2.7.2.1,2.7.2.7,2.7.2.14,2.7.2.15))) |
| D070601 | Ethylene glycol | Alcohol degradation | 1.1.1.77 1.2.1.21 |
| D070801 | Phytol | Alcohol degradation | 1.1.1.1 1.2.1.3 6.2.1.3 1.3.1.38 |
| D070901 | Polyvinyl alcohol | Alcohol degradation | 1.1.2.6 |
| D050101 | Serine | Amino acid degradation | 4.3.1.17,4.3.1.18 |
| D050201 | Threonine | Amino acid degradation | 4.3.1.19 |
| D050301 | Cysteine | Amino acid degradation | 4.4.1.1,4.4.1.28 |
| D050302 | Cysteine | Amino acid degradation | 2.6.1.3 2.8.1.2 |
| D050401 | Methionine | Amino acid degradation | 4.4.1.11 |
| D050501 | Valine | Amino acid degradation | 2.6.1.42 1.2.1.25 1.3.8.5 4.2.1.150 3.1.2.4 1.1.1.31 1.2.1.27 |
| D050502 | Valine | Amino acid degradation | 2.6.1.42 4.1.1.72 1.1.1.1 |
| D050601 | Isoleucine | Amino acid degradation | 2.6.1.42 1.2.1.25 1.3.8.5 4.2.1.150 1.1.1.178 2.3.1.16 |
| D050602 | Isoleucine | Amino acid degradation | 2.6.1.42 1.2.7.7 |
| D050701 | Leucine | Amino acid degradation | K00826 (((K00166+K00167),K11381)+K09699+K00382) (K00253,K00249) (K01968+K01969) (K05607,K13766) K01640 |
| D050801 | Lysine | Amino acid degradation | 4.1.1.18 2.6.1.82 1.2.1.19 2.6.1.48 1.2.1.20 1.14.11.64 1.1.5.13 |
| D050802 | Lysine | Amino acid degradation | 1.13.12.2 3.5.1.30 1.6.1.48 1.2.1.20 2.8.3.13 |
| D050803 | Lysine | Amino acid degradation | 2.6.1.36 1.2.1.31 |
| D050804 | Lysine | Amino acid degradation | 4.1.1.18 2.6.1.82 1.2.1.19 2.6.1.48 1.2.1.20 2.8.3.13 |
| D050805 | Lysine | Amino acid degradation | K01582 K09251 K00137 K07250 K00135 K15737 K15736 |
| D050806 | Lysine | Amino acid degradation | K00468 K01506 (K14268,K07250) K00135 ((K15737 K15736),(K01041 K00252 (K01692,K01825,K01782) (K01825,K01782) K00626)) |
| D050901 | Arginine | Amino acid degradation | 4.1.1.19 3.5.3.11 |
| D050902 | Arginine | Amino acid degradation | 4.1.1.19 3.5.3.12 3.5.1.53 |
| D050903 | Arginine | Amino acid degradation | 1.13.12.1 3.5.1.4 3.5.3.7 |
| D050904 | Arginine | Amino acid degradation | K01476 K01581 |
| D050905 | Arginine | Amino acid degradation | K00613 K00542 K00933 |
| D050906 | Arginine | Amino acid degradation | (K01583,K01584,K01585,K02626) K01480 K01611 K00797 |
| D051001 | Proline | Amino acid degradation | 5.1.1.4 1.21.4.1 |
| D051101 | Glutamate | Amino acid degradation | 1.4.1.2,1.4.1.3 |
| D051102 | Glutamate | Amino acid degradation | 2.6.1.1 4.3.1.1 |
| D051103 | Glutamate | Amino acid degradation | 1.4.1.2 1.1.1.399 2.8.3.12 4.2.1.167 7.2.4.5 |
| D051104 | Glutamate | Amino acid degradation | 5.4.99.1 4.3.1.2 4.2.1.34 4.1.3.22 |
| D051105 | Glutamate | Amino acid degradation | 4.1.1.15 |
| D051201 | Histidine | Amino acid degradation | K01745 K01712 K01468 (K01479,K00603,K13990,(K05603 K01458)) |
| D051301 | Tryptophan | Amino acid degradation | (K00453,K00463) (K01432,K14263,K07130) K00486 K01556 K00452 K03392 (K10217,K23234) |
| D051302 | Tryptophan | Amino acid degradation | 4.1.99.1 |
| D051601 | Beta-alanine | Amino acid degradation | 2.6.1.18 1.2.1.18 |
| D051602 | Beta-alanine | Amino acid degradation | 2.6.1.120 1.1.1.298 |
| D051701 | Ornithine | Amino acid degradation | 4.1.1.17 ((2.6.1.82 1.2.1.19),(6.3.1.11 1.4.3.M3 1.2.1.00 3.5.1.94)) |
| D051801 | GABA | Amino acid degradation | 2.6.1.19 (1.2.1.24,1.2.1.16) |
| D051802 | GABA | Amino acid degradation | 2.6.1.19 1.1.1.61 2.8.3.M6 4.2.1.120 1.3.1.109 (2.8.3.1,2.8.3.8) |
| D090101 | Penicillin | Antibiotic degradation | K18698,K18699,K18796,K18767,K18797,K19097,K19317,K18768,K18970,K19316,K22346,K18795,K19218,K19217,K17836,K18766 |
| D090201 | Carbapenem | Antibiotic degradation | K17837,K18782,K18781,K18780,K19099,K19216 |
| D090301 | Cephalosporin | Antibiotic degradation | K19095,K19096,K19100,K19101,K19214,K19215,K20319,K20320,K01467 |
| D090401 | Oxacillin | Antibiotic degradation | K17838,K18790,K18791,K19098,K18792,K19213,K21276,K18793,K18971,K22352,K19209,K18976,K18973,K18794,K18972,K21277,K19210,K19211,K19212,K22335,K19319,K22331,K22351,K19320,K19318,K19321,K19322,K21266,K22334,K22333,K22332 |
| D090501 | Streptogramin | Antibiotic degradation | K19349,K19350 |
| D090601 | Fosfomycin | Antibiotic degradation | K21252 |
| D090701 | Tetracycline | Antibiotic degradation | K08151,K08168,K18214,K18218,K18220,K18221 |
| D090801 | Macrolide | Antibiotic degradation | K06979,K08217,K18230,K18231,K21251 |
| D091001 | Chloramphenicol | Antibiotic degradation | K00638,K08160,K18552,K18553,K18554,K19271 |
| D091101 | Lincosamide | Antibiotic degradation | K18236,K19349,K19350,K19545 |
| D091201 | Streptothricin | Antibiotic degradation | K19273,K20816 |
| D010101 | Triglyceride | Lipid degradation | (K01046,K12298,K16816,K13534,K14073,K14074,K14075,K14076,K22283,K14452,K22284,K14674,K14675,K17900) (K01054,K25824) |
| D010102 | Triglyceride | Lipid degradation | (3.1.1.3,3.1.1.34) (3.1.1.34,3.1.1.79,3.1.1.116) (3.1.1.23,3.1.1.79) |
| D010201 | Fatty acid | Lipid degradation | (K01897,K15013) (K00232,K00249,K00255,K06445,K09479) (((K01692,K07511,K13767) (K00022,K07516)),K01825,K01782,K07514,K07515,K10527) (K00632,K07508,K07509,K07513) |
| D010301 | Oleate | Lipid degradation | 6.2.1.3 1.3.8.8 4.2.1.17 1.1.1.35 2.3.1.16 1.3.8.8 4.2.1.17 1.1.1.35 2.3.1.16 1.3.8.8 4.2.1.17 (1.1.1.35,1.1.1.211) 2.3.1.16 5.3.3.8 4.2.1.74 |
| D010401 | Dicarboxylic acids | Lipid degradation | 6.2.1.5 1.3.8.7 4.2.1.17 1.1.1.35 2.3.1.174 |
| D060101 | Nitrate | Nitrogen compound degradation | ((K00370+K00371+K00374),(K02567+K02568)) ((K00362+K00363),(K03385+K15876)) |
| D060102 | Nitrate | Nitrogen compound degradation | 1.7.5.1 1.7.2.1 1.7.2.5 1.7.2.4 |
| D060103 | Nitrate | Nitrogen compound degradation | 1.9.6.1 1.7.2.2 |
| D060105 | Nitrate | Nitrogen compound degradation | 1.7.7.2 1.7.7.1 |
| D060201 | Urea | Nitrogen compound degradation | 6.3.4.6 3.5.1.54 |
| D060202 | Urea | Nitrogen compound degradation | 3.5.1.5 |
| D060301 | Urate | Nitrogen compound degradation | 1.7.3.3 3.5.2.17 4.1.1.97 |
| D060302 | Urate | Nitrogen compound degradation | 1.14.13.113 3.5.2.17 4.1.1.97 |
| D060401 | GlcNAc | Nitrogen compound degradation | 2.7.1.59 3.5.1.25 3.5.99.6 |
| D060402 | GlcNAc | Nitrogen compound degradation | 3.5.1.25 3.5.99.6 |
| D060601 | Allantoin | Nitrogen compound degradation | 3.5.2.5 3.5.3.9 3.5.3.26 (1.1.1.350,1.1.1.154) 2.1.3.5 |
| D060602 | Allantoin | Nitrogen compound degradation | 3.5.2.5 3.5.3.4 4.3.2.3 |
| D060603 | Allantoin | Nitrogen compound degradation | 3.5.2.5 3.5.3.9 3.5.3.26 4.3.2.3 |
| D060701 | Creatinine | Nitrogen compound degradation | 3.5.4.21 3.5.2.14 3.5.1.59 1.5.3.1 |
| D060801 | Betaine | Nitrogen compound degradation | 2.1.1.5 1.5.3.10 (1.5.3.24,1.5.3.1) |
| D060901 | L-carnitine | Nitrogen compound degradation | 1.14.13.239 1.2.1.4 1.1.1.38 |
| D061001 | Methylamine | Nitrogen compound degradation | 1.4.9.1 |
| D061002 | Methylamine | Nitrogen compound degradation | 6.3.4.12 2.1.1.21 1.5.99.5 |
| D061101 | Phenylethylamine | Nitrogen compound degradation | (1.4.3.4,1.4.3.21) 1.2.1.39 |
| D061201 | Hypotaurine | Nitrogen compound degradation | 2.6.1.77 1.2.1.3 |
| D061301 | Taurine | Nitrogen compound degradation | 2.6.1.77 |
| D061303 | Taurine | Nitrogen compound degradation | 2.5.1.55 |
| D020101 | Cellulose | Polysaccharide degradation | (3.2.1.4,3.2.1.176,3.2.1.132,3.2.1.73) (3.2.1.176,3.2.1.4,3.2.1.14) (1.14.99.54,1.14.99.56,1.14.99.53) (1.14.99.54,1.14.99.53) 1.14.99.54 (1.14.99.54,1.14.99.56) (1.14.99.54,1.14.99.56,1.14.99.53) (3.2.1.4,3.2.1.8,3.2.1.21,3.2.1.25,3.2.1.45,3.2.1.58,3.2.1.73,3.2.1.74,3.2.1.75,3.2.1.78,3.2.1.91,3.2.1.104,3.2.1.123,3.2.1.132,3.2.1.149,3.2.1.151,3.2.1.164,3.2.1.168,3.2.1.73,3.2.1.39,3.2.1.52,3.2.1.132,3.2.1.146) (3.2.1.4,3.2.1.91) (3.2.1.4,3.2.1.176,3.2.1.132,3.2.1.73) (3.2.1.132,3.2.1.4,3.2.1.73,3.2.1.8,3.2.1.156) (3.2.1.4,3.2.1.6,3.2.1.21,3.2.1.73,3.2.1.74,3.2.1.91,3.2.1.151,3.2.1.165) (3.2.1.8,3.2.1.32,3.2.1.4) 3.2.1.4 (3.2.1.4,3.2.1.151,3.2.1.73,2.4.1.207) (3.2.1.4,3.2.1.151) (3.2.1.4,3.2.1.151,3.2.1.78) (3.2.1.4,3.2.1.8,3.2.1.21,3.2.1.25,3.2.1.45,3.2.1.58,3.2.1.73,3.2.1.74,3.2.1.75,3.2.1.78,3.2.1.91,3.2.1.104,3.2.1.123,3.2.1.132,3.2.1.149,3.2.1.151,3.2.1.164,3.2.1.168,3.2.1.73,3.2.1.39,3.2.1.52,3.2.1.132,3.2.1.146) (3.2.1.4,3.2.1.91) (3.2.1.4,3.2.1.176,3.2.1.132,3.2.1.73) (3.2.1.132,3.2.1.4,3.2.1.73,3.2.1.8,3.2.1.156) (3.2.1.4,3.2.1.6,3.2.1.21,3.2.1.73,3.2.1.74,3.2.1.91,3.2.1.151,3.2.1.165) (3.2.1.8,3.2.1.32,3.2.1.4) 3.2.1.4 (3.2.1.4,3.2.1.151,3.2.1.73,2.4.1.207) (3.2.1.4,3.2.1.151) (3.2.1.4,3.2.1.151,3.2.1.78) |
| D020201 | Xyloglucan | Polysaccharide degradation | (3.2.1.4,3.2.1.8,3.2.1.21,3.2.1.25,3.2.1.45,3.2.1.58,3.2.1.73,3.2.1.74,3.2.1.75,3.2.1.78,3.2.1.91,3.2.1.104,3.2.1.123,3.2.1.132,3.2.1.149,3.2.1.151,3.2.1.164,3.2.1.168,3.2.1.73,3.2.1.39,3.2.1.52,3.2.1.132,3.2.1.146) (3.2.1.4,3.2.1.6,3.2.1.21,3.2.1.73,3.2.1.74,3.2.1.91,3.2.1.151,3.2.1.165) (3.2.1.4,3.2.1.151,3.2.1.73,2.4.1.207) (2.4.1.207,3.2.1.103,3.2.1.39,3.2.1.6,3.2.1.73,3.2.1.81,3.2.1.83,3.2.1.151,3.2.1.181,3.2.1.178,3.2.1.35,3.2.1.181) (3.2.1.4,3.2.1.151) (3.2.1.4,3.2.1.151,3.2.1.78) (3.2.1.176,3.2.1.4,3.2.1.14) (3.2.1.4,3.2.1.150,3.2.1.151) (1.14.99.54,1.14.99.56) (3.2.1.20,3.2.1.22,3.2.1.24,3.2.1.84,3.2.1.48,3.2.1.10,3.2.1.177,4.2.2.13,2.4.1.161) (3.2.1.37,3.2.1.55,3.2.1.8,3.2.1.99,3.2.1.145,3.2.1.146) (3.2.1.8,3.2.1.32,3.2.1.4) (3.2.1.23,3.2.1.25,3.2.1.31,3.2.1.55,3.2.1.152,3.2.1.165,3.2.1.37,3.2.1.146) |
| D020301 | Starch | Polysaccharide degradation | (3.2.1.1,3.2.1.41,2.4.1.19,3.2.1.54,3.2.1.93,3.2.1.10,3.2.1.133,3.2.1.135,3.2.1.20,3.2.1.60,3.2.1.68,3.2.1.70,3.2.1.98,3.2.1.116,2.4.1.18,5.4.99.16,2.4.1.25,2.4.1.4,2.4.1.7,3.2.1.141,5.4.99.11,5.4.99.15,3.2.1.33,2.4.99.16) 3.2.1.2 (3.2.1.1,3.2.1.22,3.2.1.41,3.2.1.54,2.4.1.18,2.4.1.25) 3.2.1.1 3.2.1.33 (3.2.1.3,3.2.1.70,3.2.1.28,2.4.1.2) (3.2.1.3,3.2.1.20,3.2.1.22) |
| D020401 | Chitin | Polysaccharide degradation | (3.2.1.14,3.2.1.17,3.2.1.96) (3.2.1.14,3.2.1.17) (3.2.1.17,4.2.2.n1,3.2.1.14) (3.2.1.17,3.2.1.96) (3.2.1.52,3.2.1.140) (3.2.1.21,3.2.1.37,3.2.1.45,3.2.1.52,3.2.1.55,3.2.1.58,3.2.1.74,3.2.1.120,3.2.1.126) (3.2.1.4,3.2.1.8,3.2.1.21,3.2.1.25,3.2.1.45,3.2.1.58,3.2.1.73,3.2.1.74,3.2.1.75,3.2.1.78,3.2.1.91,3.2.1.104,3.2.1.123,3.2.1.132,3.2.1.149,3.2.1.151,3.2.1.164,3.2.1.168,3.2.1.73,3.2.1.39,3.2.1.52,3.2.1.132,3.2.1.146) (3.2.1.52,3.2.1.35,3.2.1.169) (3.2.1.21,3.2.1.37,3.2.1.45,3.2.1.52) (1.14.99.54,1.14.99.56,1.14.99.53) (1.14.99.54,1.14.99.53) (3.1.1.72,3.5.1.41) |
| D020501 | Pectin | Polysaccharide degradation | (3.2.1.15,3.2.1.40,3.2.1.67,3.2.1.82,3.2.1.171,3.2.1.173) (4.2.2.2,4.2.2.9,4.2.2.10) (4.2.2.2,4.2.2.9) (4.2.2.2,4.2.2.9) 4.2.2.2 (4.2.2.23,4.2.2.24) 4.2.2.6 4.2.2.24 4.2.2.23 (3.2.1.40,3.2.1.174) 3.2.1.173 3.1.1.11 3.2.1.172 (3.2.1.122,3.2.1.20,3.2.1.22,3.2.1.86,3.2.1.139,3.2.1.67) 3.1.1.72 (3.2.1.23,3.2.1.25,3.2.1.31,3.2.1.55,3.2.1.152,3.2.1.165,3.2.1.37,3.2.1.146) |
| D020601 | Alpha galactan | Polysaccharide degradation | 3.2.1.49 (3.2.1.22,3.2.1.49,3.2.1.94,3.2.1.88) 3.2.1.22 (3.2.1.122,3.2.1.20,3.2.1.22,3.2.1.86,3.2.1.139,3.2.1.67) (3.2.1.20,3.2.1.22,3.2.1.24,3.2.1.84,3.2.1.48,3.2.1.10,3.2.1.177,4.2.2.13,2.4.1.161) (3.2.1.22,3.2.1.49,2.4.1.67,2.4.1.82) (3.2.1.3,3.2.1.20,3.2.1.22) |
| D020701 | Beta-galactan | Polysaccharide degradation | 3.2.1.89 (3.2.1.23,3.2.1.25,3.2.1.31,3.2.1.55,3.2.1.152,3.2.1.165,3.2.1.37,3.2.1.146) (3.2.1.23,3.2.1.165) 3.2.1.23 (3.2.1.21,3.2.1.23,3.2.1.25,3.2.1.31,3.2.1.37,3.2.1.38,3.2.1.62,3.2.1.74,3.2.1.85,3.2.1.86,3.2.1.105,3.2.1.108,3.2.1.117,3.2.1.118,3.2.1.119,3.2.1.125,3.2.1.147,3.2.1.149,3.2.1.161,3.2.1.175,3.2.1.182) (3.2.1.23,3.2.1.46) |
| D020801 | Mixed-Linkage glucans | Polysaccharide degradation | 3.2.1.71 (3.2.1.8,3.2.1.31,3.2.1.37,3.2.1.38,3.2.1.45,3.2.1.75,3.2.1.136) (3.2.1.39,3.2.1.58,3.2.1.73,3.2.1.175) (3.2.1.4,3.2.1.176,3.2.1.132,3.2.1.73) (3.2.1.132,3.2.1.4,3.2.1.73,3.2.1.8,3.2.1.156) (3.2.1.4,3.2.1.6,3.2.1.21,3.2.1.73,3.2.1.74,3.2.1.91,3.2.1.151,3.2.1.165) (3.2.1.4,3.2.1.8,3.2.1.21,3.2.1.25,3.2.1.45,3.2.1.58,3.2.1.73,3.2.1.74,3.2.1.75,3.2.1.78,3.2.1.91,3.2.1.104,3.2.1.123,3.2.1.132,3.2.1.149,3.2.1.151,3.2.1.164,3.2.1.168,3.2.1.73,3.2.1.39,3.2.1.52,3.2.1.132,3.2.1.146) (3.2.1.58,3.2.1.39) 3.2.1.39 (3.2.1.21,3.2.1.37,3.2.1.45,3.2.1.52,3.2.1.55,3.2.1.58,3.2.1.74,3.2.1.120,3.2.1.126) (2.4.1.207,3.2.1.103,3.2.1.39,3.2.1.6,3.2.1.73,3.2.1.81,3.2.1.83,3.2.1.151,3.2.1.181,3.2.1.178,3.2.1.35,3.2.1.181) (3.2.1.39,3.2.1.58,3.2.1.73,3.2.1.175) (3.2.1.58,3.2.1.39) (3.2.1.21,3.2.1.23,3.2.1.25,3.2.1.31,3.2.1.37,3.2.1.38,3.2.1.62,3.2.1.74,3.2.1.85,3.2.1.86,3.2.1.105,3.2.1.108,3.2.1.117,3.2.1.118,3.2.1.119,3.2.1.125,3.2.1.147,3.2.1.149,3.2.1.161,3.2.1.175,3.2.1.182) |
| D020901 | Xylans | Polysaccharide degradation | (3.2.1.8,3.2.1.32,3.2.1.4) (3.2.1.78,3.2.1.100,3.2.1.32,3.2.1.73) (3.2.1.132,3.2.1.4,3.2.1.73,3.2.1.8,3.2.1.156) (3.2.1.4,3.2.1.8,3.2.1.21,3.2.1.25,3.2.1.45,3.2.1.58,3.2.1.73,3.2.1.74,3.2.1.75,3.2.1.78,3.2.1.91,3.2.1.104,3.2.1.123,3.2.1.132,3.2.1.149,3.2.1.151,3.2.1.164,3.2.1.168,3.2.1.73,3.2.1.39,3.2.1.52,3.2.1.132,3.2.1.146) (3.2.1.8,3.2.1.32) (3.2.1.8,3.2.1.31,3.2.1.37,3.2.1.38,3.2.1.45,3.2.1.75,3.2.1.136) (3.2.1.102,3.2.1.8,3.2.1.8) (3.2.1.51,3.2.1.8) (3.2.1.76,3.2.1.37) (3.2.1.21,3.2.1.37,3.2.1.45,3.2.1.52,3.2.1.55,3.2.1.58,3.2.1.74,3.2.1.120,3.2.1.126) (3.2.1.20,3.2.1.22,3.2.1.24,3.2.1.84,3.2.1.48,3.2.1.10,3.2.1.177,4.2.2.13,2.4.1.161) 3.2.1.37 (3.2.1.139,3.2.1.131) |
| D021001 | Beta-mannan | Polysaccharide degradation | 3.2.1.78 (3.2.1.78,3.2.1.100,3.2.1.32,3.2.1.73) (3.2.1.4,3.2.1.8,3.2.1.21,3.2.1.25,3.2.1.45,3.2.1.58,3.2.1.73,3.2.1.74,3.2.1.75,3.2.1.78,3.2.1.91,3.2.1.104,3.2.1.123,3.2.1.132,3.2.1.149,3.2.1.151,3.2.1.164,3.2.1.168,3.2.1.73,3.2.1.39,3.2.1.52,3.2.1.132,3.2.1.146) (3.2.1.78,3.2.1.100,3.2.1.32,3.2.1.73) (2.4.1.281,2.4.1.319,2.4.1.320) 3.2.1.78 3.1.1.72 (3.2.1.23,3.2.1.25,3.2.1.31,3.2.1.55,3.2.1.152,3.2.1.165,3.2.1.37,3.2.1.146) |
| D021101 | Alpha-mannan | Polysaccharide degradation | 3.2.1.130 (3.2.1.101,3.2.1.20) (3.2.1.101,3.2.1.20) (3.2.1.113,3.2.1.24) (3.2.1.24,3.2.1.113,3.2.1.114,3.2.1.170) (3.2.1.106,3.2.1.84,3.2.1.20,3.2.1.170,3.2.1.208) 3.2.1.113 (2.4.1.281,2.4.1.319,2.4.1.320) |
| D021201 | Arabinan | Polysaccharide degradation | (3.2.1.37,3.2.1.55,3.2.1.8,3.2.1.99,3.2.1.145,3.2.1.146) (3.2.1.11,3.2.1.57,3.2.1.95) (3.2.1.37,3.2.1.55,3.2.1.8,3.2.1.99,3.2.1.145,3.2.1.146) (3.2.1.4,3.2.1.8,3.2.1.37,3.2.1.55,3.2.1.73) (3.2.1.21,3.2.1.37,3.2.1.45,3.2.1.52,3.2.1.55,3.2.1.58,3.2.1.74,3.2.1.120,3.2.1.126) (3.2.1.55,3.2.1.37) 3.2.1.55 |
| D021301 | Mucin | Polysaccharide degradation | 3.2.1.97 (3.2.1.22,3.2.1.49,3.2.1.94,3.2.1.88) 3.2.1.49 (2.4.1.211,2.4.1.247) (3.2.1.20,3.2.1.22,3.2.1.24,3.2.1.84,3.2.1.48,3.2.1.10,3.2.1.177,4.2.2.13,2.4.1.161) (3.2.1.22,3.2.1.49,2.4.1.67,2.4.1.82) |
| D030101 | Lactose | Sugar degradation | 3.2.1.85 5.3.1.26 2.7.1.144 4.1.2.40 |
| D030201 | Sucrose | Sugar degradation | 2.7.1.211 3.2.1.48 2.7.1.4 |
| D030302 | D-Apiose | Sugar degradation | 1.1.1.420 3.1.1.115 |
| D030401 | D-Arabinose | Sugar degradation | 5.3.1.3 2.7.1.47 |
| D030402 | D-Arabinose | Sugar degradation | 5.3.1.3 2.7.1.51 4.1.2.17 1.2.1.21 |
| D030501 | D-Mannose | Sugar degradation | 2.7.1.191 5.3.1.8 |
| D030502 | D-Mannose | Sugar degradation | 2.7.1.7 5.3.1.8 |
| D030601 | D-Xylose | Sugar degradation | 5.3.1.5 2.7.1.17 |
| D030602 | D-Xylose | Sugar degradation | 1.1.1.9 2.7.1.17 |
| D030603 | D-Xylose | Sugar degradation | 1.1.1.424 3.1.1.68 4.2.1.82 4.2.1.141 1.2.1.26 |
| D030604 | D-Xylose | Sugar degradation | 1.1.1.359 3.1.1.110 4.2.1.82 4.1.2.28 1.1.1.26 2.3.3.9 |
| D030605 | D-Xylose | Sugar degradation | 1.1.1.175 3.1.1.110 4.2.1.82 4.2.1.1.141 1.2.1.26 |
| D030701 | L-Fucose | Sugar degradation | 5.1.3.29 5.3.1.25 2.7.1.51 4.1.2.17 |
| D030702 | L-Fucose | Sugar degradation | 5.1.3.29 4.2.1.68 1.1.1.M68 3.7.1.26 |
| D030801 | L-Rhamnose | Sugar degradation | 5.1.3.32 5.3.1.14 2.7.1.5 4.1.2.19 |
| D030802 | L-Rhamnose | Sugar degradation | (1.1.1.173,1.1.1.378) 3.1.1.65 4.2.1.90 4.1.2.53 |
| D030803 | L-Rhamnose | Sugar degradation | (1.1.1.173,1.1.1.378) 3.1.1.65 4.2.1.90 1.1.1.401 3.7.1.26 |
| D030901 | Galactose | Sugar degradation | K01785 K00849 K00965 K01784 |
| D031001 | NeuAc | Sugar degradation | 4.1.3.3 5.1.3.8 |
| D031002 | NeuAc | Sugar degradation | 4.1.3.3 2.7.1.60 5.1.3.9 |
| D080101 | Toluene | Xenobiotic degradation | (K15760+K15761+K15763+K15764) K00055 K00141 |
| D080103 | Toluene | Xenobiotic degradation | K07540 (K07543+K07544) K07545 K07546 (K07547+K07548) (K07549+K07550) |
| D080201 | Xylene | Xenobiotic degradation | (K15757+K15758) K00055 K00141 |
| D080402 | Benzene | Xenobiotic degradation | K16249+K16243+K16244+K16242+K16245+K16246 |
| D080501 | Benzoate | Xenobiotic degradation | (K05549+K05550+K05784) K05783 |
| D080502 | Benzoate | Xenobiotic degradation | K04116 K04117 K07534 K07535 K07536 |
| D080601 | Anthranilate | Xenobiotic degradation | (K05599+K05600+K11311),(K16319+K16320+K18248+K18249) |
| D080701 | Catechol | Xenobiotic degradation | K03381 K01856 K03464 (K01055,K14727) |
| D080702 | Catechol | Xenobiotic degradation | (K00446,K07104) ((K10217 K01821 K01617),K10216) (K18364,K02554) (K18365,K01666) (K18366,K04073) |
| D080801 | Cumate | Xenobiotic degradation | (K10619+K16303+K16304+K18227) K10620 K10621 K10622 K10623 |
| D080901 | Biphenyl | Xenobiotic degradation | (K08689+K15750+K18087+K18088) K08690 K00462 K10222 |
| D081001 | Carbazole | Xenobiotic degradation | K15751 (K15754+K15755) K15756 |
| D081101 | Benzoyl-CoA | Xenobiotic degradation | ((K04112+K04113+K04114+K04115),(K19515+K19516)) K07537 K07538 K07539 |
| D081201 | Naphthalene | Xenobiotic degradation | (K14579+K14580+K14578+K14581) K14582 K14583 K14584 K14585 K00152 |
| D081301 | Salicylate | Xenobiotic degradation | K18242+K18243+K14578+K14581 |
| D081401 | Terephthalate | Xenobiotic degradation | (K18074+K18075+K18077) K18076 |
| D081501 | Phthalate | Xenobiotic degradation | (K18068+K18069) K18067 K04102 |
| D081601 | Phenylacetate | Xenobiotic degradation | K01912 (K02609+K02610+K02611+K02612+K02613) K15866 K02618 K02615 K01692 K00074 |
| D081701 | Trans-cinnamate | Xenobiotic degradation | (((K05708+K05709+K05710+K00529) K05711),K05712) K05713 K05714 K02554 K01666 K04073 |
| D081801 | Caffeine | Xenobiotic degradation | K21722 K21723 K21724 |
| D081901 | Mercury | Xenobiotic degradation | 4.99.1.2 1.16.1.1 |
| B020401 | Serine | Amino acid biosynthesis | K00058 K00831 (K01079,K02203,K22305,K25528) |
| B020501 | Threonine | Amino acid biosynthesis | (K00928,K12524,K12525,K12526) K00133 (K00003,K12524,K12525) (K00872,K02204,K02203) K01733 |
| B020601 | Cysteine | Amino acid biosynthesis | (K00640,K23304) (K01738,K13034,K17069) |
| B020602 | Cysteine | Amino acid biosynthesis | (K01697,K10150) K01758 |
| B020603 | Cysteine | Amino acid biosynthesis | K00789 K17462 K01243 K07173 K17216 K17217 |
| B020701 | Methionine | Amino acid biosynthesis | (K00928,K12524,K12525) K00133 (K00003,K12524,K12525) (K00651,K00641) K01739 (K01760,K14155) (K00548,K24042,K00549) |
| B020801 | Valine | Amino acid biosynthesis | (K01652+(K01653,K11258)) K00053 K01687 K00826 |
| B020901 | Isoleucine | Amino acid biosynthesis | (K01703+K01704) K00052 |
| B020902 | Isoleucine | Amino acid biosynthesis | (K17989,K01754) (K01652+(K01653,K11258)) K00053 K01687 K00826 |
| B021001 | Leucine | Amino acid biosynthesis | K01649 (K01702,(K01703+K01704)) K00052 |
| B021101 | Lysine | Amino acid biosynthesis | (K00928,K12524,K12525,K12526) K00133 K01714 K00215 K00674 (K00821,K14267) K01439 K01778 (K01586,K12526) |
| B021102 | Lysine | Amino acid biosynthesis | K00928 K00133 K01714 K00215 K05822 K00841 K05823 K01778 K01586 |
| B021103 | Lysine | Amino acid biosynthesis | (K00928,K12524,K12525,K12526) K00133 K01714 K00215 K03340 (K01586,K12526) |
| B021104 | Lysine | Amino acid biosynthesis | (K00928,K12524,K12525,K12526) K00133 K01714 K00215 K10206 K01778 (K01586,K12526) |
| B021105 | Lysine | Amino acid biosynthesis | K01655 ((K17450 K01705),(K16792+K16793)) K05824 |
| B021106 | Lysine | Amino acid biosynthesis | K05827 K05828 K05829 K05830 K05831 |
| B021201 | Arginine | Amino acid biosynthesis | K00611 K01940 (K01755,K14681) |
| B021202 | Arginine | Amino acid biosynthesis | K22478 K00145 K00821 K09065 K01438 K01940 K01755 |
| B021301 | Proline | Amino acid biosynthesis | ((K00931 K00147),K12657) K00286 |
| B021401 | Glutamate | Amino acid biosynthesis | K00673 K01484 K00840 K06447 K05526 |
| B021402 | Glutamate | Amino acid biosynthesis | K01745 K01712 K01468 (K01479,K00603,K13990,(K05603 K01458)) |
| B021501 | Histidine | Amino acid biosynthesis | K00765 ((K01523 K01496),K11755,K14152) (K01814,K24017) ((K02501+K02500),K01663) ((K01693 K00817 (K04486,K05602,K18649)),(K01089 K00817)) (K00013,K14152) |
| B021601 | Tryptophan | Amino acid biosynthesis | ((((K01657+K01658),K13503,K13501,K01656) K00766),K13497) (((K01817,K24017) (K01656,K01609)),K13498,K13501) ((K01695+(K01696,K06001)),K01694) |
| B021701 | Phenylalanine | Amino acid biosynthesis | (((K01850,K04092,K14187,K04093,K04516,K06208,K06209) (K01713,K04518,K05359)),K14170) (K00832,K00838) |
| B021801 | Tyrosine | Amino acid biosynthesis | (((K01850,K04092,K14170,K04093,K04516,K06208,K06209) (K04517,K00211)),K14187) (K00832,K00838) |
| B021802 | Tyrosine | Amino acid biosynthesis | (K01850,K04092,K14170) (K00832,K15849) (K00220,K24018,K15227) |
| B021901 | GABA | Amino acid biosynthesis | K09470 K09471 K09472 K09473 |
| B022001 | Beta-alanine | Amino acid biosynthesis | (K00207,(K17722+K17723)) K01464 (K01431,K06016) |
| B022002 | Beta-alanine | Amino acid biosynthesis | 6.2.1.17 1.3.8.1 4.2.1.116 3.1.2.4 1.1.159 2.6.1.18 |
| B022101 | Ornithine | Amino acid biosynthesis | (K00618,K00619,K14681,K14682,K00620,K22477,K22478) (((K00930,K22478) K00145),K12659) (K00818,K00821) (K01438,K14677,K00620) |
| B022102 | Ornithine | Amino acid biosynthesis | K19412 K05828 K05829 K05830 K05831 |
| B022103 | Ornithine | Amino acid biosynthesis | 2.3.1.1 2.7.2.8 1.2.1.38 2.6.1.11 3.5.1.16 |
| B030201 | Betaine | Amino acid derivative biosynthesis | 1.1.99.1 1.2.1.8 |
| B030202 | Betaine | Amino acid derivative biosynthesis | 2.1.1.156 2.1.1.157 |
| B030301 | Ectoine | Amino acid derivative biosynthesis | K00928 K00133 K00836 K06718 K06720 |
| B030701 | Spermidine | Amino acid derivative biosynthesis | (K01583,K01584,K01585,K02626) K01480 |
| B030901 | Putrescine | Amino acid derivative biosynthesis | K01476 K01581 |
| B031001 | Tryptamine | Amino acid derivative biosynthesis | (4.1.1.28,4.1.1.105) |
| B080101 | Salicylate | Aromatic compound biosynthesis | 5.4.4.2 4.2.99.21 |
| B080201 | Gallate | Aromatic compound biosynthesis | 4.2.1.10 |
| B080301 | Chorismate | Aromatic compound biosynthesis | 4.2.1.10 1.1.1.25 2.7.1.71 2.5.1.19 4.2.3.5 |
| B080302 | Chorismate | Aromatic compound biosynthesis | 2.5.1.54 3.2.3.4 4.2.1.10 1.1.1.25 2.7.1.71 2.5.1.19 4.2.3.5 |
| B080303 | Chorismate | Aromatic compound biosynthesis | 2.7.2.4 1.2.1.11 2.2.1.10 1.4.1.24 4.2.1.10 1.1.1.25 2.7.1.71 2.5.1.19 4.2.3.5 |
| B080404 | Dipicolinate | Aromatic compound biosynthesis | 2.7.2.4 1.2.1.11 4.3.3.7 |
| B060101 | Succinate | Organic anion biosynthesis | (K01647,K05942) (K01681,K01682) (K00031,K00030) ((((K00164+K00658),K01616)+K00382),(K00174+K00175)) ((K01902+K01903),(K01899+K01900),K18118) ((K00234+K00235+K00236+(K00237,K25801)),(K00239+K00240+K00241),(K00244+K00245+K00246)) (K01676,K01679,(K01677+K01678)) (K00026,K00025,K00024,K00116) |
| B060102 | Succinate | Organic anion biosynthesis | ((((K00164+K00658),K01616)+K00382),K00174) ((K01902+K01903),(K01899+K01900),K18118) ((K00234+K00235+K00236+(K00237,K25801)),(K00239+K00240+K00241),(K00244+K00245+K00246)) (K01676,K01679,(K01677+K01678)) (K00026,K00025,K00024,K00116) |
| B060103 | Succinate | Organic anion biosynthesis | K01580 (K13524,K07250,K00823,K16871) (K00135,K00139,K17761) |
| B060104 | Succinate | Organic anion biosynthesis | (K00169+K00170+K00171+K00172) K01007 K01595 K00024 (K01677+K01678) (K00239+K00240) (K01902+K01903) (K15038,K15017) K14465 (K14467,K18861) K14534 K15016 K00626 |
| B060105 | Succinate | Organic anion biosynthesis | (K02160+K01961+K01962+K01963) K14468 K14469 K15052 K05606 (K01847,(K01848+K01849)) (K14471+K14472) (K00239+K00240+K00241) K01679 K08691 K14449 K14470 K09709 |
| B060106 | Succinate | Organic anion biosynthesis | (K00169+K00170+K00171+K00172) (K01959+K01960) K00024 (K01677+K01678) (K18209+K18210) (K01902+K01903) (K00174+K00175+K00176+K00177) |
| B060201 | Fumarate | Organic anion biosynthesis | (K01647,K05942) (K01681,K01682) (K00031,K00030) ((((K00164+K00658),K01616)+K00382),(K00174+K00175)) ((K01902+K01903),(K01899+K01900),K18118) ((K00234+K00235+K00236+(K00237,K25801)),(K00239+K00240+K00241),(K00244+K00245+K00246)) (K01676,K01679,(K01677+K01678)) (K00026,K00025,K00024,K00116) |
| B060202 | Fumarate | Organic anion biosynthesis | ((((K00164+K00658),K01616)+K00382),K00174) ((K01902+K01903),(K01899+K01900),K18118) ((K00234+K00235+K00236+(K00237,K25801)),(K00239+K00240+K00241),(K00244+K00245+K00246)) (K01676,K01679,(K01677+K01678)) (K00026,K00025,K00024,K00116) |
| B060203 | Fumarate | Organic anion biosynthesis | K01948 K00611 K01940 (K01755,K14681) K01476 |
| B060204 | Fumarate | Organic anion biosynthesis | (K00815,K00838,K00832,K03334) K00457 K00451 K01800 (K01555,K16171) |
| B060205 | Fumarate | Organic anion biosynthesis | K00241+(K00242,K18859,K18860)+K00239+K00240 |
| B060206 | Fumarate | Organic anion biosynthesis | K00244+K00245+K00246+K00247 |
| B060207 | Fumarate | Organic anion biosynthesis | (K00169+K00170+K00171+K00172) K01007 K01595 K00024 (K01677+K01678) (K00239+K00240) (K01902+K01903) (K15038,K15017) K14465 (K14467,K18861) K14534 K15016 K00626 |
| B060208 | Fumarate | Organic anion biosynthesis | (K02160+K01961+K01962+K01963) K14468 K14469 K15052 K05606 (K01847,(K01848+K01849)) (K14471+K14472) (K00239+K00240+K00241) K01679 K08691 K14449 K14470 K09709 |
| B060209 | Fumarate | Organic anion biosynthesis | (K00169+K00170+K00171+K00172) (K01959+K01960) K00024 (K01677+K01678) (K18209+K18210) (K01902+K01903) (K00174+K00175+K00176+K00177) |
| B060210 | Fumarate | Organic anion biosynthesis | (K18029+K18030) K14974 K18028 K15357 K13995 K01799 |
| B060211 | Fumarate | Organic anion biosynthesis | K00611 K01940 (K01755,K14681) |
| B060212 | Fumarate | Organic anion biosynthesis | K22478 K00145 K00821 K09065 K01438 K01940 K01755 |
| B060213 | Fumarate | Organic anion biosynthesis | 2.6.1.1,2.6.1.5,2.6.1.27,2.6.1.57 1.13.11.27 1.13.11.5 5.2.1.2 3.7.1.2 |
| B060301 | Citrate | Organic anion biosynthesis | (K01647,K05942) (K01681,K01682) (K00031,K00030) ((((K00164+K00658),K01616)+K00382),(K00174+K00175)) ((K01902+K01903),(K01899+K01900),K18118) ((K00234+K00235+K00236+(K00237,K25801)),(K00239+K00240+K00241),(K00244+K00245+K00246)) (K01676,K01679,(K01677+K01678)) (K00026,K00025,K00024,K00116) |
| B060302 | Citrate | Organic anion biosynthesis | (K01647,K05942) (K01681,K01682) (K00031,K00030) |
| B060303 | Citrate | Organic anion biosynthesis | K01647 (K01681,K01682) K01637 (K01638,K19282) (K00026,K00025,K00024) |
| B060304 | Citrate | Organic anion biosynthesis | K01647 K01681 K00031 K00261 (K19268+K01846) K04835 K19280 K14449 K19281 K19282 K00024 |
| B040101 | Acetate | SCFA biosynthesis | (K00625,K13788,K15024) K00925 |
| B040103 | Acetate | SCFA biosynthesis | K01067 |
| B040104 | Acetate | SCFA biosynthesis | 5.4.3.2 5.4.3.3 1.4.1.11 2.3.1.247 1.3.1.109 2.8.3.9 (2.3.1.9,2.3.1.16) 2.3.1.8 (2.7.2.1,2.7.2.15) |
| B040105 | Acetate | SCFA biosynthesis | 1.21.4.2 (2.7.2.1,2.7.2.15) |
| B040106 | Acetate | SCFA biosynthesis | (1.2.7.1,1.2.1.104) 2.3.1.8 (2.7.2.1,2.7.2.15) |
| B040201 | Butyrate | SCFA biosynthesis | 1.2.7.1 (2.3.1.9,2.3.1.16) 1.1.1.35 4.2.1.150 1.3.1.109 2.3.1.19 (2.7.2.7,2.7.2.14) |
| B040202 | Butyrate | SCFA biosynthesis | 2.3.1.8 (2.7.2.1,2.7.2.15) (2.8.3.1,2.8.3.8) |
| B040203 | Butyrate | SCFA biosynthesis | (2.3.1.9,2.3.1.16) 1.1.1.36 4.2.1.55 1.3.1.109 (2.8.3.1,2.8.3.8) |
| B040204 | Butyrate | SCFA biosynthesis | 1.4.1.2 1.1.1.399 2.8.3.12 4.2.1.167 7.2.4.5 1.3.1.109 (2.8.3.1,2.8.3.8) |
| B040205 | Butyrate | SCFA biosynthesis | 5.4.3.2 5.4.3.3 1.4.1.11 2.3.1.247 1.3.1.109 2.8.3.9 |
| B040206 | Butyrate | SCFA biosynthesis | 2.8.3.18 1.2.1.76 1.1.1.61 2.8.3.M6 4.2.1.120 1.3.1.109 (2.8.3.1,2.8.3.8) |
| B040207 | Butyrate | SCFA biosynthesis | 1.4.1.2 1.1.1.399 2.8.3.12 4.2.1.167 7.2.4.5 1.3.1.109 (2.8.3.1,2.8.3.8) |
| B040208 | Butyrate | SCFA biosynthesis | 1.4.1.2 1.1.1.399 2.8.3.12 4.2.1.167 7.2.4.5 1.3.1.109 (2.8.3.1,2.8.3.8) |
| B040301 | Propionate | SCFA biosynthesis | (4.2.1.28,1.1.1.1) 1.2.1.87 2.3.1.222 (2.7.2.1,2.7.2.7,2.7.2.14,2.7.2.15) |
| B040302 | Propionate | SCFA biosynthesis | 4.3.1.19 2.3.1.222 (2.7.2.1,2.7.2.7,2.7.2.14,2.7.2.15) |
| B040304 | Propionate | SCFA biosynthesis | 2.1.3.1 1.1.1.37 4.2.1.2 1.3.5.1 2.8.3.27 |
| B040305 | Propionate | SCFA biosynthesis | 2.8.3.1 4.2.1.54 1.3.1.95 2.8.3.1 |
| B040306 | Propionate | SCFA biosynthesis | 2.6.1.2 1.1.1.28 2.8.3.1 4.2.1.54 1.3.1.95 2.8.3.1 |
| B070101 | Thiamine (B1) | Vitamin biosynthesis | (((K03148+K03154) K03151),(K03150 K03149)) K03147 ((K00941 K00788),K14153,K21219) K00946 |
| B070102 | Thiamine (B1) | Vitamin biosynthesis | (((K03148+K03154) K03151),(K03153 K03149 K10810)) K03147 K00941 K00788 K00946 |
| B070103 | Thiamine (B1) | Vitamin biosynthesis | (K22699,K03147) ((K00941 (K00788,K21220)),K21219) K00946 |
| B070104 | Thiamine (B1) | Vitamin biosynthesis | (K00941 K00788),K14153,K21219 |
| B070201 | Riboflavin (B2) | Vitamin biosynthesis | (((K01497,K14652) ((K01498 K00082),K11752) (K22912,K20860,K20861,K20862,K21063,K21064)),(K02858,K14652)) K00794 K00793 ((K20884 K22949),K11753) |
| B070301 | Niacin (B3) | Vitamin biosynthesis | 3.6.1.22 (3.2.2.6,3.2.2.4) 3.4.1.19 |
| B070401 | Pantothenate (B5) | Vitamin biosynthesis | ((K00826 K00606 K00077),K01579) (K01918,K13799) |
| B070402 | Pantothenate (B5) | Vitamin biosynthesis | ((K00606 K00077),(K13367 K00128)) K01918 |
| B070501 | Pyridoxal-P (B6) | Vitamin biosynthesis | K03472 K03473 K00831 K00097 K03474 K00275 |
| B070502 | Pyridoxal-P (B6) | Vitamin biosynthesis | K06215 K08681 |
| B070601 | Biotin (B7) | Vitamin biosynthesis | K00652 (((K00833,K19563) K01935),K19562) K01012 |
| B070602 | Biotin (B7) | Vitamin biosynthesis | K00652 K25570 K01935 K01012 |
| B070603 | Biotin (B7) | Vitamin biosynthesis | K16593 K00652 K19563 K01935 K01012 |
| B070604 | Biotin (B7) | Vitamin biosynthesis | K01906 K00652 (K00833,K19563) K01935 K01012 |
| B070701 | Tetrahydrofolate (B9) | Vitamin biosynthesis | (K01495,K09007,K22391) (K01077,K01113,(K08310,K19965)) ((K13939,((K13940,(K01633 K00950)) K00796)),(K01633 K13941)) (K11754,K20457) (K00287,K13998) |
| B070702 | Tetrahydrofolate (B9) | Vitamin biosynthesis | K14652 K22100 K01633 K13941 K22099 K00287 |
| B070801 | Cobalamin (B12) | Vitamin biosynthesis | (K02302,((K02303,K13542) (K02304,K24866))) (K02190,K03795,K22011) K03394 (K05934,K13541,K21479) K05936 (K02189,K13541) K02188 K05895 ((K02191 K03399),K00595) K06042 K02224 |
| B070802 | Cobalamin (B12) | Vitamin biosynthesis | (K02303,K13542) (K03394,K13540) K02229 (K05934,K13540,K13541) K05936 K02228 K05895 K00595 K06042 K02224 K02230+K09882+K09883 |
| B070803 | Cobalamin (B12) | Vitamin biosynthesis | (K00798,K19221) K02232 (K02225,K02227) K02231 K00768 (K02226,K22316) K02233 |
| B070901 | Tocopherol/tocotorienol (E) | Vitamin biosynthesis | K09833 (K12502,K18534) K09834 K05928 |
| B071001 | Phylloquinone (K1) | Vitamin biosynthesis | ((K02552 K02551 K08680 K02549),K14759) (K01911,K14760) K01661 (K19222,K12073) K23094 K17872 K23095 |
| B071101 | Menaquinone (K2) | Vitamin biosynthesis | K02552 K02551 K08680 K02549 K01911 K01661 K19222 K02548 K03183 |
| B071102 | Menaquinone (K2) | Vitamin biosynthesis | K11782 K18285 (K18286,K20810) K11783 K11784 K11785 |
| B071103 | Menaquinone (K2) | Vitamin biosynthesis | K11782 K18285 K18284 K11784 K11785 |
| B071201 | Ubiquinone (Q10) | Vitamin biosynthesis | (K03181,K18240) K03179 (K03182+K03186) K18800 K00568 K03185 K03183 (K03184,K06134) K00568 |
| B071202 | Ubiquinone (Q10) | Vitamin biosynthesis | K06125 K06126 K00591 K06127 K06134 K00591 |

**Supplementary table 2:** Final mixed linear models for assessment of within species Alpha and Beta diversities.

| **Model name** | **Diversity components** | **Diversity type** | **Model equation** |
| --- | --- | --- | --- |
| Crocidura1 | neutral | alpha | diversity~treatment,  random=~1\|Cage |
| Crocidura2 | functional | alpha | diversity~treatment,  random=~1\|Cage/Mouse_ID,  correlation=days |
| Crocidura3 | phylogenetic | alpha | diversity~treatment,  random=~1\|Cage/Mouse_ID,  correlation=days |
| Crocidura3 | neutral | beta | dissimilarity~treatment_pair*sex,  random=~1\|Cage/Mouse_ID |
| Crocidura4 | functional | beta | dissimilarity~treatment_pair,  random=~1\|Cage/Mouse_ID,  correlation=days |
| Crocidura6 | phylogenetic | beta | dissimilarity~treatment_pair*sex,  random=~1\|Cage/Mouse_ID,  correlation=days |
| Apodemus1 | neutral | alpha | diversity~treatment,  random=~1\|Cage/Mouse_ID,  correlation=days |
| Apodemus2 | functional | alpha | diversity~treatment,  random=~1\|Cage/Mouse_ID,  correlation=days |
| Apodemus3 | phylogenetic | alpha | diversity~treatment,  random=~1\|Cage/Mouse_ID,  correlation=days |
| Apodemus4 | neutral | beta | dissimilarity~treatment_pair,  random=~1\|Mouse_ID,  correlation=days |
| Apodemus5 | functional | beta | dissimilarity~treatment_pair,  random=~1\|cage/Mouse_ID,  correlation=days |
| Apodemus6 | phylogenetic | beta | dissimilarity~treatment_pair,  random=~1\|Cage/Mouse_ID,  correlation=days |

Supplementary figures **Supplementary figure 1:** Housing conditions used throughout the experiment. Natural elements were used, collected from local areas where the animals were caught. Enrichment material consisted of sticks, stones and moss. As well as a small aerated house to bring the nesting material in.

**
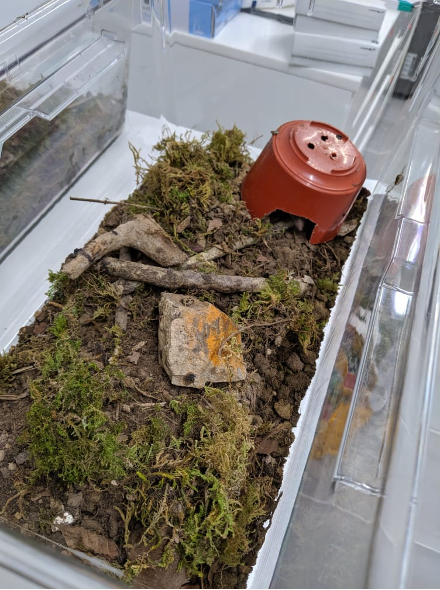
**

**Supplementary figure 2a**: Metabolic capacity index values for each of the measured pathways in *Apodemus sylvaticus*.


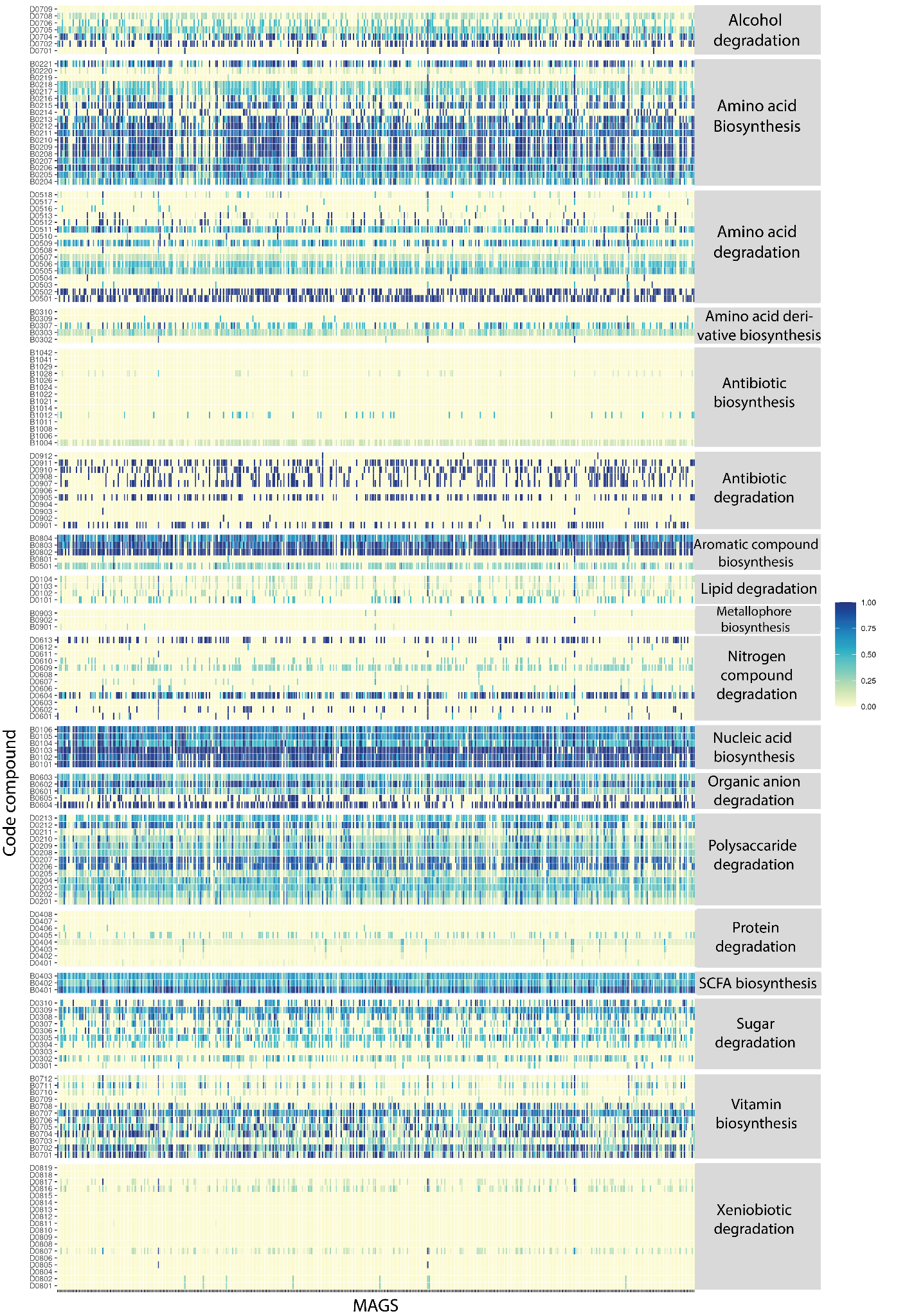


**Supplementary figure 2b**: Metabolic capacity index values for each of the measured pathways in *Crocidura russula*.


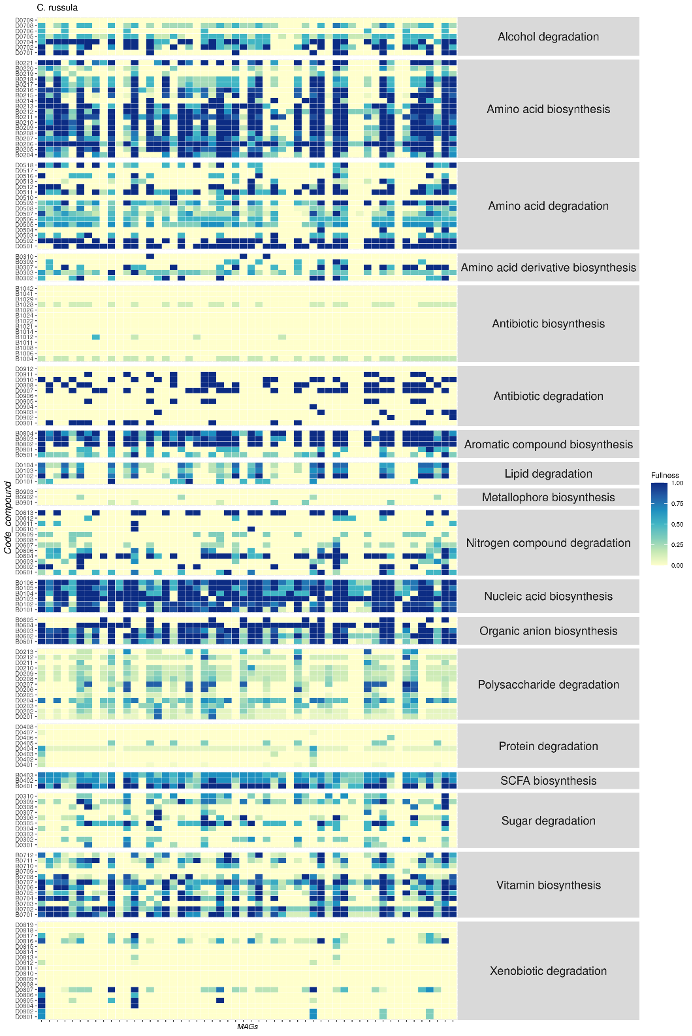


**Supplementary figure 3:** PCA depicting the functional dynamics and MAG abundance dynamics across the consecutive disturbances across time in *C. russula*. The ordination was conducted on the community MCI values and MAG abundances predicted by the Hmsc model for each time point. With the objective of facilitating visual representation, just the 20% of the MAGs with highest correlation with the PC1 axis of the ordination are shown. MAGs and functional MCIs located on the same side of the ordination indicate similar temporal dynamics (i.e. when a MAG increased or decreased in a time step, the functional MCI also tended to increase or decrease in the same direction), whereas MAGs and functional MCIs located at opposite sides indicate antagonistic dynamics (i.e. when a MAG increased or decreased in a time step, the functional MCI tended to decrease or increase, following the opposite direction).
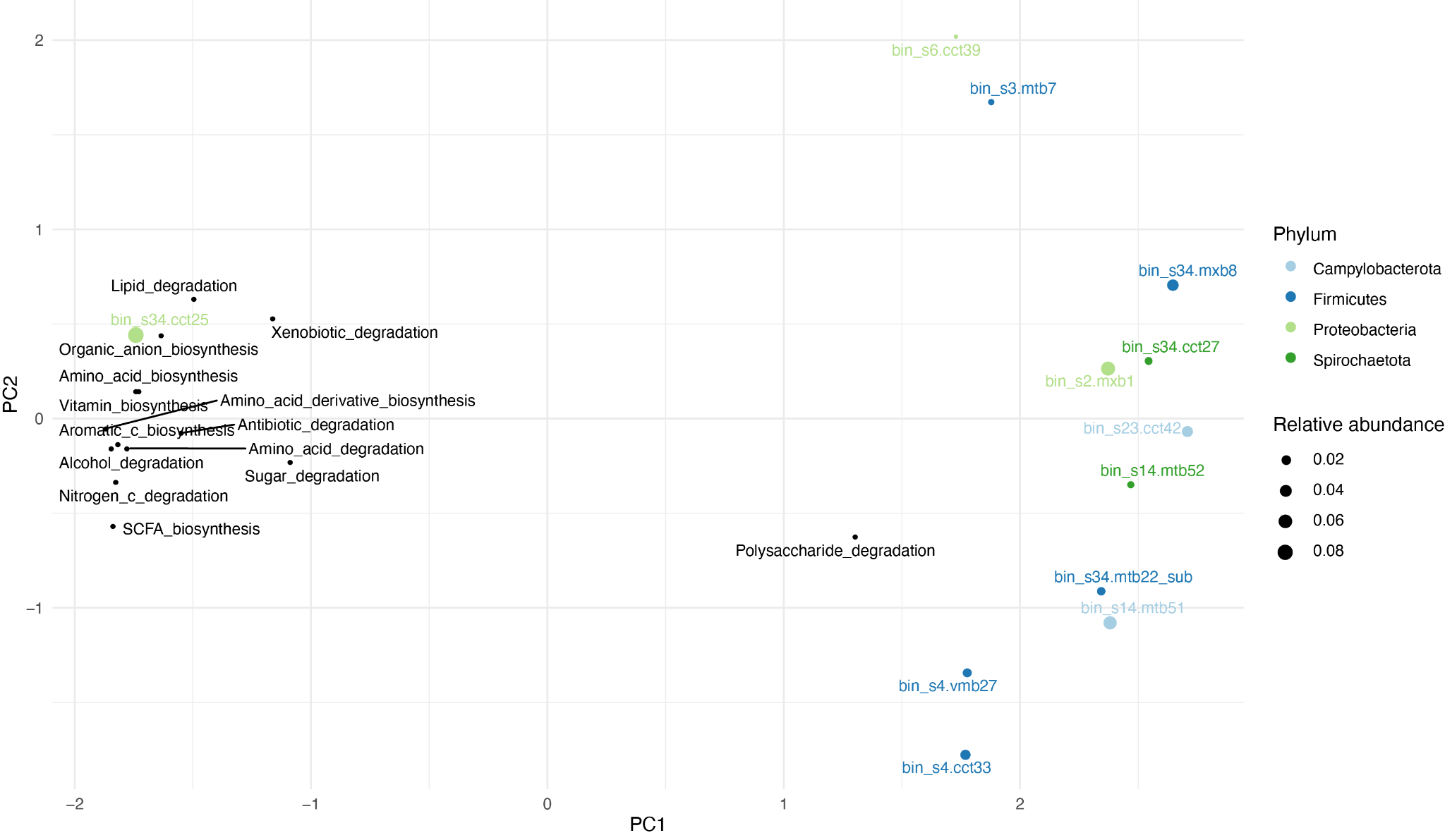

Supplement: Supplemental material — Supplemental tables and figures. [file mbio.01606-23-s0001.docx]
